# ABT1 modifies SMARD1 pathology via interactions with IGHMBP2 and stimulation of ATPase and helicase activity

**DOI:** 10.1172/jci.insight.164608

**Published:** 2023-01-24

**Authors:** Gangadhar P. Vadla, Sara M. Ricardez Hernandez, Jiude Mao, Mona O. Garro-Kacher, Zachary C. Lorson, Ronin P. Rice, Sarah A. Hansen, Christian L. Lorson, Kamal Singh, Monique A. Lorson

**Affiliations:** 1 Department of Veterinary Pathobiology, College of Veterinary Medicine, and; 2 Bond Life Sciences Center, University of Missouri, Columbia, Missouri, USA.

**Keywords:** Genetics, Neuroscience, Genetic diseases, Molecular genetics, Neurological disorders

## Abstract

SMA with respiratory distress type 1 (SMARD1) and Charcot-Marie-Tooth type 2S (CMT2S) are results of mutations in immunoglobulin *mu* DNA binding protein 2 (*IGHMBP2*). IGHMBP2 is a UPF1-like helicase with proposed roles in several cellular processes, including translation. This study examines activator of basal transcription 1 (ABT1), a modifier of SMARD1-nmd disease pathology. Microscale thermophoresis and dynamic light scattering demonstrate that IGHMBP2 and ABT1 proteins directly interact with high affinity. The association of ABT1 with IGHMBP2 significantly increases the ATPase and helicase activity as well as the processivity of IGHMBP2. The IGHMBP2/ABT1 complex interacts with the 47S pre-rRNA 5′ external transcribed spacer and U3 small nucleolar RNA (snoRNA), suggesting that the IGHMBP2/ABT1 complex is important for pre-rRNA processing. Intracerebroventricular injection of scAAV9-*Abt1* decreases FVB-*Ighmbp2^nmd/nmd^* disease pathology, significantly increases lifespan, and substantially decreases neuromuscular junction denervation. To our knowledge, ABT1 is the first disease-modifying gene identified for SMARD1. We provide a mechanism proposing that ABT1 decreases disease pathology in FVB-*Ighmbp2^nmd/nmd^* mutants by optimizing IGHMBP2 biochemical activity (ATPase and helicase activity). Our studies provide insight into SMARD1 pathogenesis, suggesting that ABT1 modifies IGHMBP2 activity as a means to regulate pre-rRNA processing.

## Graphical abstract

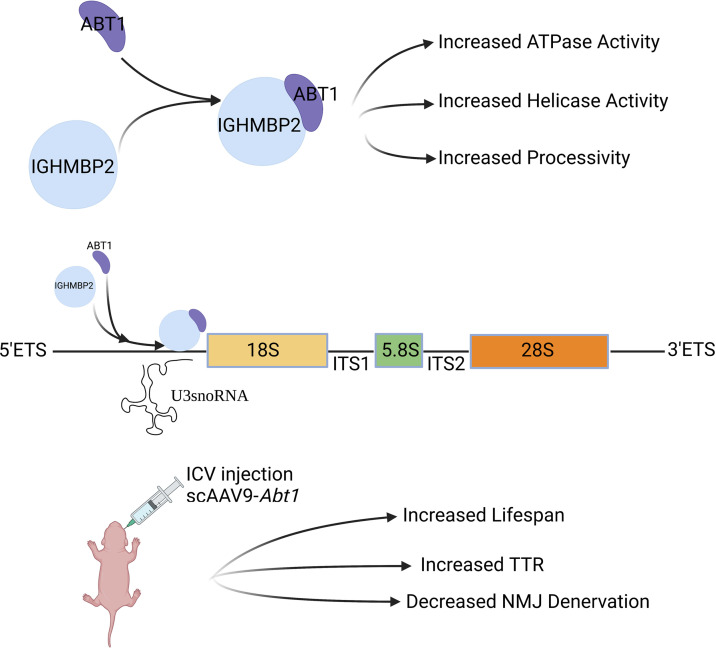

## Introduction

Homozygous or compound heterozygous mutations in immunoglobulin *mu* DNA binding protein 2 (*IGHMBP2)* result in 2 distinct diseases: SMA with respiratory distress type 1 (SMARD1) and Charcot-Marie-Tooth type 2S (CMT2S). The first major SMARD1 clinical symptom, respiratory complication, is due to diaphragmatic paralysis that typically manifests at 6 weeks to 13 months of age (1–4). SMARD1 is characterized by distal lower limb muscle atrophy, followed by proximal muscle weakness that is a result of degeneration of the anterior horn cells. Intrauterine growth retardation, autonomic nervous system, and sensory defects are present (1–7).

CMT represents a large group of disorders associated with neuropathies defined by mutations in many genes. CMT2S is a progressive motor sensory axonal neuropathy caused by certain *IGHMBP2* mutations. Patients with CMT2S present with a slow but progressive distal muscle weakness and atrophy that manifests later than SMARD1, and there is no respiratory impairment. CMT2S clinical presentations include delayed milestones, difficulty walking, sensory and autonomic dysfunctions, gait impairment, and foot drop (8–12).

IGHMBP2 is a Up-frameshift 1–like (UPF1-like) family of SF1 helicases that includes UPF1, IGHMBP2, Senataxin, and Moloney leukemia virus 10 (MOV10). UPF1 is an RNA helicase required for nonsense-mediated decay (13, 14). Senataxin is required for resolving R-Loop structures and transcription termination and, when mutated, results in ALS or ataxia with oculomotor apraxia (15, 16). MOV10 functions in miRNA-dependent regulation (17–19). The UPF1-like helicases contain 2 RecA-like domains (domains 1A and 2A) and 2 subdomains (domains 1B and 1C). Conformational changes in subdomains 1B and 1C occur upon RNA binding (20, 21). In addition to the helicase domain, IGHMBP2 contains R3H and zinc finger domains. Studies suggest that the R3H domain in IGHMBP2 regulates RNA binding and stimulates ATPase activity (21, 22). Recombinant IGHMBP2 ATPase activity is stimulated by nucleic acid, and unwinding occurs in a 5′ to 3′ direction (23). In vitro studies show that IGHMBP2 self-associates and interacts with a subset of small RNAs including tRNA^Tyr^, TFIIIC220, proteins of the large and small ribosomal subunit, 5S, 18S and 28S rRNAs, Reptin, Pontin, and activator of basal transcription 1 (ABT1) (23, 24). IGHMBP2 has proposed roles in immunoglobulin class switching, pre-mRNA maturation, transcription regulation, and translation (22, 23, 25, 26). It is unknown what biochemical processes, when IGHMBP2 is mutated, result in disease, and the distinctions that lead to SMARD1 or CMT2S are also unknown.

The original SMARD1 mouse model, called B6-*nmd*^2J^, originated from a spontaneous mutation identified in the Jackson Laboratory and was later discovered as an A-to-G mutation in intron 4 of *Ighmbp2*. This mutation creates a cryptic splice site that disrupts the normal splicing pattern of the *Ighmbp2* transcript, resulting in aberrant splicing in ~75%–80% of the *Ighmbp2* mRNAs (27, 28). The mutant transcript includes 23 additional nucleotides and a premature stop codon; full-length (FL) IGHMBP2 protein is reduced in these mice (28, 29). The lifespan of B6-*nmd*^2J^ mice is quite variable (weeks to several months), with weight gain typically past P22 (28–30). Phenotypic characterization includes hindlimb muscle weakness that proceeds to the forelimb and trunk muscles. By 3 weeks of age, B6-*nmd*^2J^ mice show weakness and retrenchment of their hindlimbs and are unable to complete time-to-right (TTR) and hindlimb splay (HLS) assays (27–29). Mice are paralyzed by 5 weeks of age and are unable to complete rotarod assays (29). Motor neuron loss is not apparent at 5 days, but by 10 days, there is a reduction of motor neurons by ~40%, followed by a plateau and then a significant reduction in motor neurons (~28% surviving) by weeks 12–14 (29, 31). PND10 B6-*nmd*^2J^ mice demonstrate little motor end plate denervation in the gastrocnemius; however, denervation increases as disease severity increases (29). End plate denervation is not observed in the diaphragm throughout the disease process (29, 31). Unlike SMARD1 patients, respiratory distress in B6-*nmd*^2J^ mice is largely absent (29). We generated a FVB-*Ighmbp2^+/nmd^* mouse that contains the same A-to-G mutation in intron 4 of *Ighmbp2*. FVB-*nmd* lifespan is less variable (17–21 days) and while mutant mice initially gain weight, there is a rapid weight and motor function decline (32, 33). FVB-*nmd* mutant mice demonstrate the same muscle weakness and hindlimb contractures as B6-*nmd*^2J^ mutant mice; however, disease pathology occurs earlier (32, 33).

Previously, a 166 kb CAST BAC clone (CH26-27k3) was shown to modify the B6-*nmd*^2J^ mutant phenotype (24). BAC-27k3 contains 2 coding genes, *Abt1* and zinc finger protein 322a (*Zfp322a*), 1 noncoding expressed sequence tag (EST), and 24 tRNA genes. In humans, the syntenic region contains 4 tRNA^Tyr^ genes and *ABT1*. In mice expressing the genetic modifier, neither *Ighmbp2* splicing nor IGHMBP2 protein levels were restored to WT. Motor neuron degeneration was significantly reduced in B6-*nmd*^2J^ mutant mice expressing the genetic modifier. When FLAG-IGHMBP2 and myc-ABT1 were cotransfected into HEK 293T cells, the proteins coimmunoprecipitated, demonstrating an association between ABT1 and IGHMBP2 (24).

ABT1 was first described as a protein that binds TATA-binding protein and activates basal transcription (34). ABT1 is conserved through species from human to yeast and contains an RNA recognition motif (RRM). Esf2p, a *Saccharomyces cerevisiae* homolog of ABT1, associates with the 5′ external transcribed spacer (ETS), U3 small nucleolar RNA (snoRNA), and proteins associated with the 90S preribosomal complex (35). Depletion of Esf2p inhibited cleavage of the 35S pre-rRNA at the A_0_ through A_2_ sites and SSU processome assembly (35). The Esf2p C-terminal domain associates with the RNA helicase Dbp8, and this association enhances the ATPase activity of Dbp8 in 18S rRNA synthesis (36). Furthermore, when ABT1 is reduced using siRNAs transfected into HeLa cells, 47S, 34S, and 30S pre-rRNA intermediates are increased (37).

Based on the studies outlined above, we were interested in addressing 2 questions: (a) do IGHMBP2 and ABT1 interact directly, and if so, what impact does this association have on IGHMBP2 biochemical function? And (b) does ABT1 modify the SMARD1 phenotype? We used FVB-*Ighmbp2^nmd/nmd^* mice, since their lifespan is shortened and less variable — 2 characteristics important in evaluating whether modifying properties are present. Toward this end, we demonstrate direct, high-affinity binding of ABT1 to IGHMBP2. ABT1 binding increases the ATPase and helicase activity and the processivity of IGHMBP2. We demonstrate that the IGHMBP2/ABT1 complex associates with the 47S pre-rRNA 5′ ETS and U3 snoRNA, suggesting that the IGHMBP2/ABT1 complex is important in pre-rRNA processing. When scAAV9-*Abt1* is delivered to FVB-*Ighmbp2^nmd/nmd^* mice via i.c.v. injection on P2, lifespan is extended, neuromuscular junction (NMJ) denervation is reduced, and ABT1 protein levels are increased. These studies identify ABT1 as the first modifier of SMARD1 pathology, and we propose that ABT1 associates with IGHMBP2 to regulate IGHMBP2 activities that directly impact disease severity.

## Results

### ABT1 directly associates with IGHMBP2 with a high binding affinity.

Previous studies show that ABT1 associated with IGHMBP2 through immunoprecipitation from HEK293T cell lysates (24). To determine whether the IGHMBP2/ABT1 interaction was direct, ABT1 was synthesized and cloned into pGEX-6P1 and IGHMBP2 into pET32b expression constructs. ABT1 and IGHMBP2 purified protein was generated. The pGEX-ABT1 predicted size was ~57 kDa, while the pET32-IGHMBP2 predicted size was ~124 kDa.

**Figure 1:**
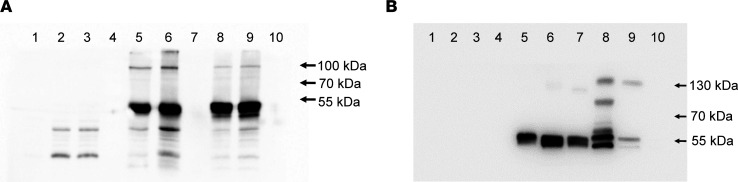
IGHMBP2 and ABT1 coimmunoprecipitate. (**A**) IGHMBP2 and ABT1 coimmunoprecipitation using anti-IGHMBP2 antibodies for Western blot. Lane 1 shows A/G beads alone, lane 2 shows A/G beads of ABT1 preclear lysate, lane 3 shows A/G beads of IGHMBP2 + ABT1 preclear lysate, lane 4 shows ABT1 lysate IP with anti-GST antibodies, lane 5 shows IGHMBP2 + ABT1 IP with anti-His antibodies, lane 6 shows IGHMBP2 + ABT1 IP with anti-IGHMBP2 antibodies, lane 7 shows protein marker, lane 8 shows IGHMBP2 + ABT1 IP with anti-ABT1 antibodies, lane 9 shows IGHMBP2 + ABT1 IP with anti-GST antibodies, and lane 10 shows IGHMBP2 + ABT1 IP with IP serum (1:20 dilution) (negative control). (**B**) IGHMBP2 and ABT1 coimmunoprecipitation using anti-ABT1 antibodies for Western blot. Lane 1 shows protein marker, lane 2 shows A/G beads alone, lane 3 shows A/G beads of ABT1 preclear lysate, lane 4 shows A/G beads of IGHMBP2 + ABT1 preclear lysate, lane 5 shows ABT1 lysate IP with anti-GST antibodies, lane 6 shows IGHMBP2 + ABT1 IP with anti-His antibodies, lane 7 shows IGHMBP2 + ABT1 IP with anti-IGHMBP2 antibodies, lane 8 shows IGHMBP2 + ABT1 IP with anti-GST antibodies, lane 9 shows IGHMBP2 + ABT1 IP with anti-ABT1 antibodies, and lane 10 shows IGHMBP2 + ABT1 IP with IP serum (1:20 dilution) (negative control). ABT1, 31 kDa ABT1 + ~26 kDa GST; IGHMBP2, 110 kDa IGHMBP2 + ~14 kDa thioredoxin–6***×*** His.

ABT1 and IGHMBP2 purified proteins were analyzed by immunoprecipitation and Western blot (Figure 1A). When pET32b-IGHMBP2 and GST-ABT1 were immunoprecipitated with His, IGHMBP2, ABT1, or GST antibodies and then analyzed by Western blot with IGHMBP2 antibody, protein at the predicted mass (~124 kDa) was identified (Figure 1A). Beads alone, precleared lysates, and the negative controls were negative for reactivity (Figure 1A). When the same samples were incubated with the ABT1 antibody, protein at the predicted mass was detected (Figure 1B). Additionally, immunoprecipitation of ABT1 with GST antibodies and Western blot with ABT1 antibody reacted similarly (Figure 1B). Beads alone, preclear lysates, and the negative control were all negative for reactivity (Figure 1B).

**Figure 2:**
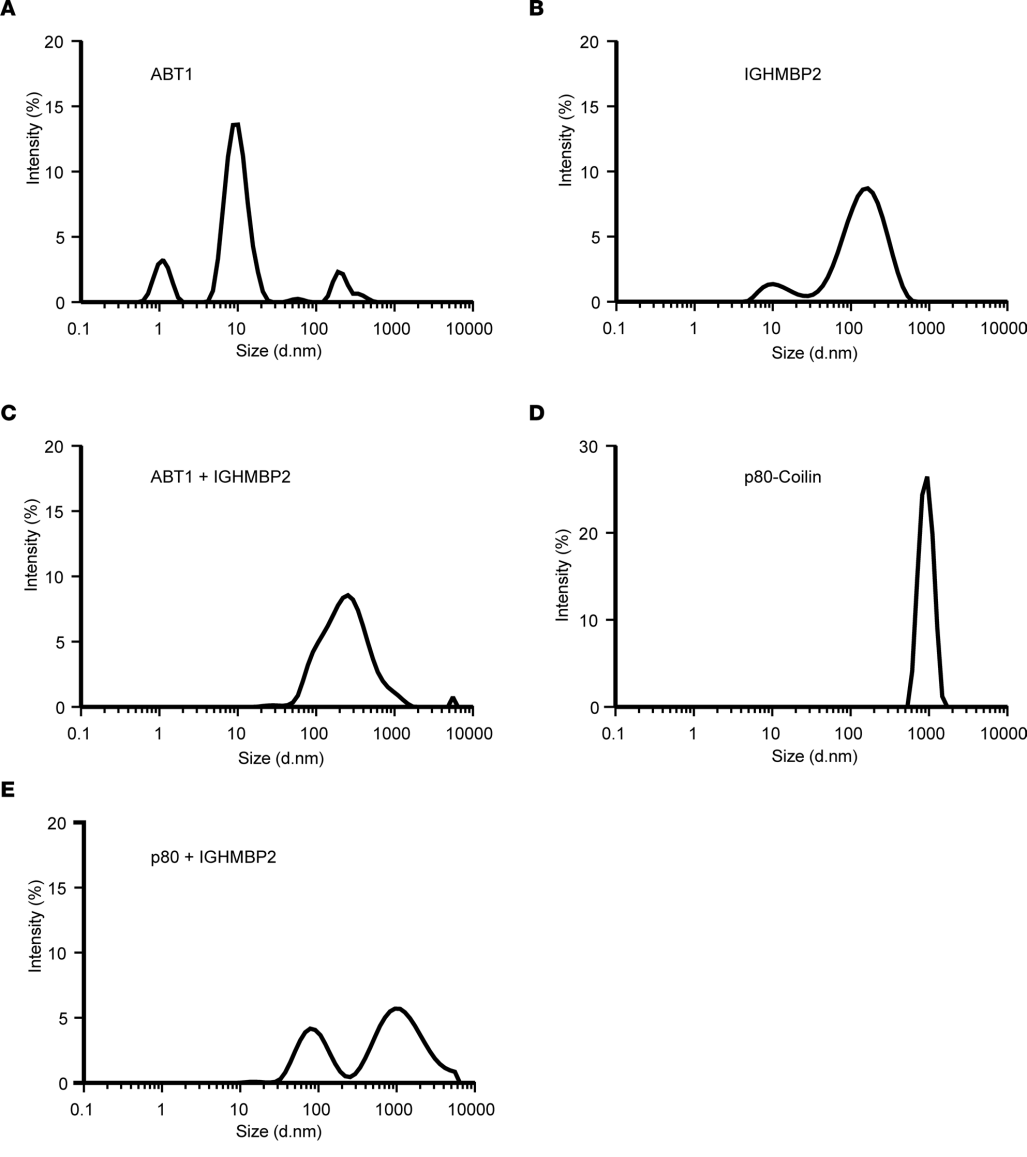
ABT1 associates with IGHMBP2. (**A**–**D**) Graphical representation of DLS physiochemical characteristics by intensity size. (**A**) Purified ABT1 (mean peak intensity was 13.6 with SD 1.38). (**B**) Purified IGHMBP2 (mean peak intensity was 8.59 with SD 0.47). (**C**) ABT1 and IGHMBP2 incubated 1:1 (mean peak intensity was 8.55 with SD 0.47). (**D**) Purified p80 (mean peak intensity was 26.47 with SD 0.61). (**E**) p80 and IGHMBP2 incubated 1:1 (mean peak intensity peak 1 was 7.37 with SD 12.76, mean peak intensity peak 2 was 7.96 with SD 7.09) serves as the nonspecific binding control. Each protein was used at 100 nm. Experiments were repeated 3 independent times for 9 measurements.

To examine the binding interaction of ABT1 with IGHMBP2, quantitative dynamic light scattering (DLS) was performed (Figure 2). DLS is widely used to determine the size of the particle — in this case, a protein — by measuring the rate of Brownian motion and the diffusion rate (38). The smaller the particle, the more rapid the diffusion rate. The intensity distribution is measured according to the scatter intensity of each particle fraction. DLS analysis of ABT1 purified protein shows 1 large primary peak and 2 smaller peaks, suggesting a largely homogeneous protein of ~10 nm (Figure 2A). DLS analysis of IGHMBP2 purified protein demonstrates a large and homogenous protein of ~110 nm (Figure 2B). When ABT1 and IGHMBP2 purified proteins are combined in a 1:1 ratio, a single peak of ~120 nm is measured that represents the association of IGHMBP2 and ABT1 (Figure 2C). p80 (Coilin) alone and the association between p80 and IGHMBP2 served as negative controls (Figure 2, D and E). p80 is a scaffold protein that accumulates in Cajal bodies and is essential for the formation/maintenance of Cajal bodies (39). Binding between p80 and IGHMBP2 did not occur, as determined by 2 independent peaks that represent each protein independently (Figure 2E). When the stoichiometry of ABT1 to IGHMBP2 was examined, it is estimated that the ratio of IGHMBP2/ABT1 is 1:1 (Supplemental Figure 1; supplemental material available online with this article; https://doi.org/10.1172/jci.insight.164608DS1). These studies confirm the homogeneity of the proteins examined and demonstrate that ABT1 directly associates with IGHMBP2.

**Figure 3:**
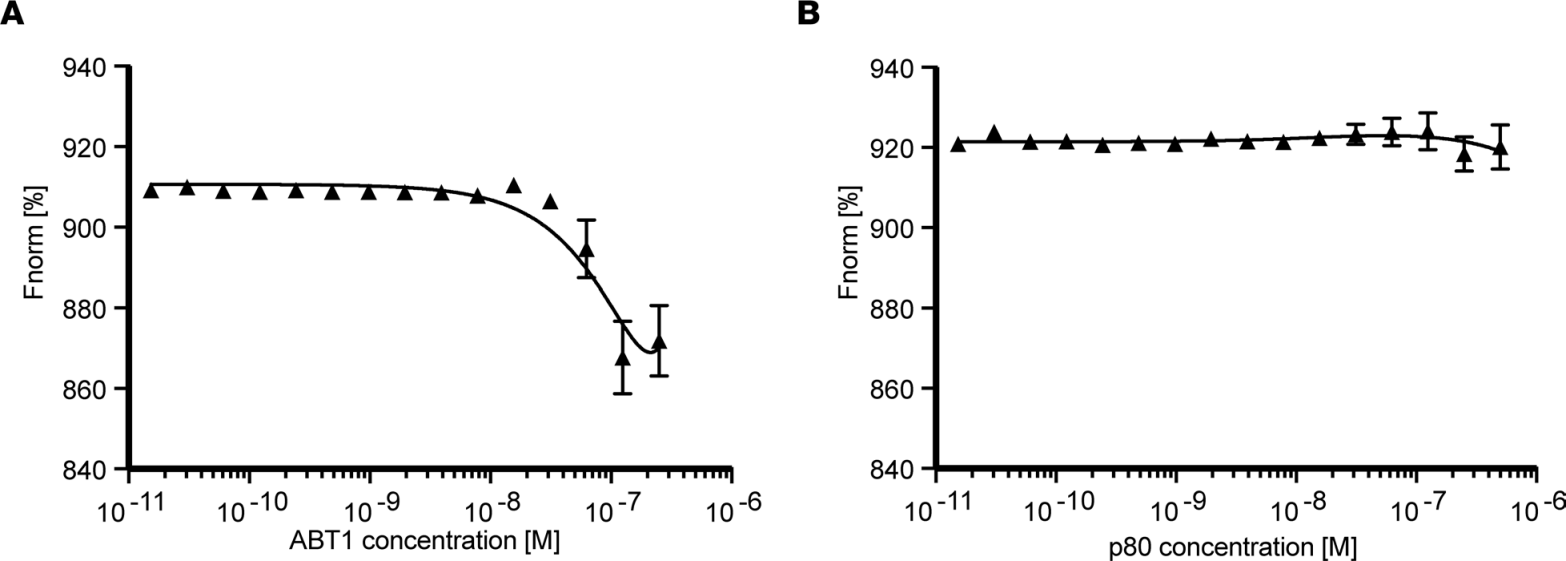
ABT1 binds IGHMBP2 with a high affinity. Representative binding isotherms generated from microscale thermophoresis (MST) with normalized fluorescence (Fnorm[%]) plotted. IGHMBP2 is RED-Tris-NTA labeled. (**A**) IGHMBP2 (100 nM) and increasing concentrations of ABT1 (1 μM to 1.53 ***×*** 10^5^ μM). The binding affinity was calculated at *K_D_* = 52 nM. (**B**) IGHMBP2 (100 nM) and increasing concentrations of p80 (1 μM to 1.53 ***×*** 10^5^ μM) (negative control). The data represent mean values from 3 independent experiments and 9 readings; data are shown as mean ± SD. The data represent mean values from 3 independent experiments and 9 readings; data are shown as mean ± SD. The MST data were analyzed with the MO.Affinity Analysis software and fit to a quadratic equation using nonlinear regression to determine the *K_D_* value.

To determine the affinity of the ABT1/IGHMBP2 binding reaction, microscale thermophoresis (MST) was performed using a titration series of purified ABT1 (1 μM to 1.53 ***×*** 10^5^ μM), while IGHMBP2 was kept at a constant concentration (100n M) (Figure 3). When increasing concentrations of ABT1 were analyzed in the presence of IGHMBP2, the binding affinity increased with increasing ABT1 concentrations (Figure 3A). The change in the thermophoretic signal demonstrates that ABT1 bound to IGHMBP2 with a binding affinity of ~52 nM (Figure 3A). To demonstrate the specificity of ABT1/IGHMBP2 binding, the binding reaction of p80 with IGHMBP2 was examined using similar titration and incubation conditions (Figure 3B). Increasing concentrations of p80 incubated with IGHMBP2 demonstrated baseline fluorescence and confirmed p80 and IGHMBP2 did not directly associate with a stable, measurable binding affinity (Figure 3B).

### Binding of ABT1 to IGHMBP2 increases ATPase and helicase activity and the processivity of IGHMBP2.

**Figure 4:**
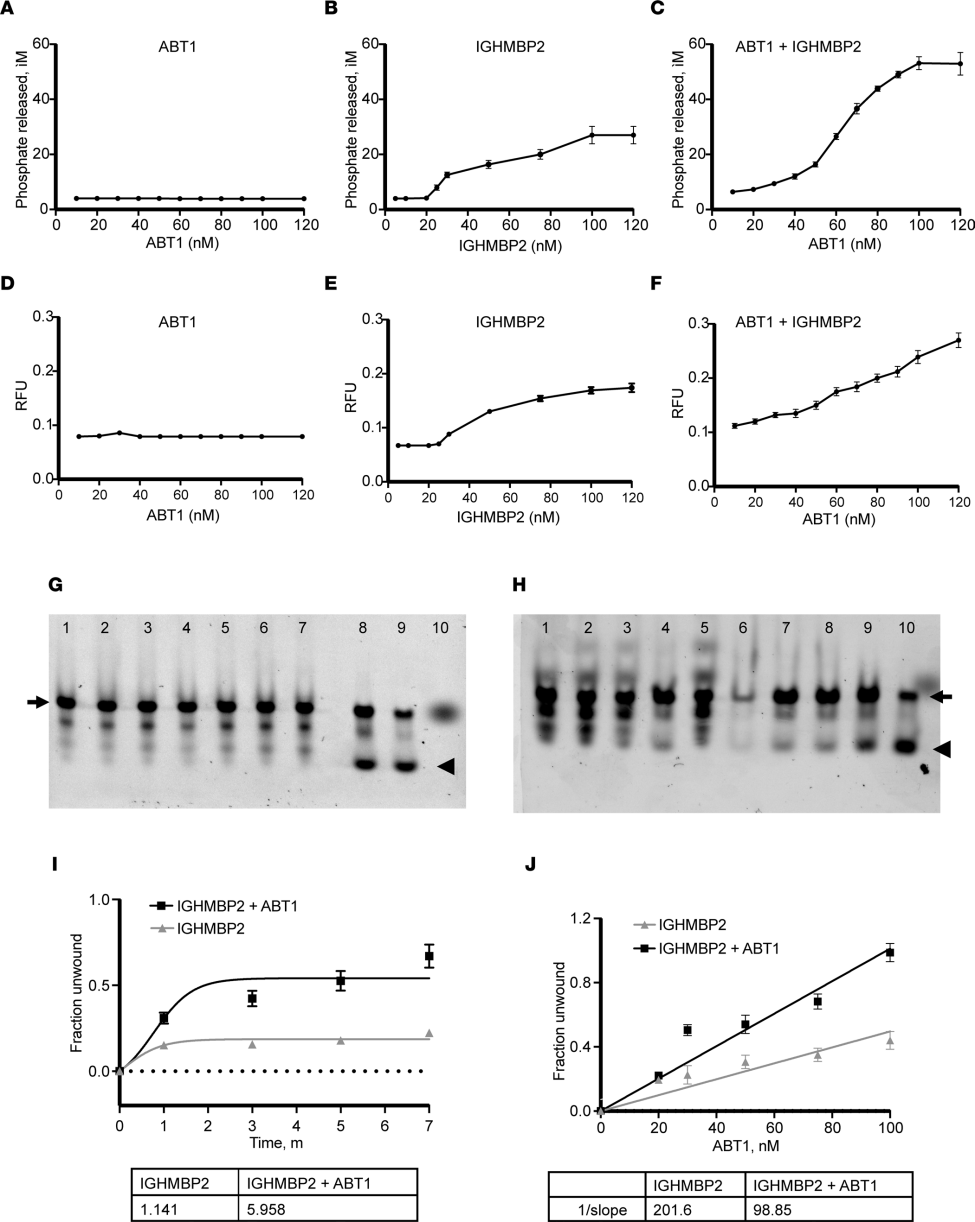
The association of ABT1 with IGHMBP2 increases the ATPase and helicase activity as well as the processivity of IGHMBP2. (**A**) ATPase activity measured with increasing concentrations of ABT1. (**B**) Increasing concentrations of IGHMBP2. (**C**) IGHMBP2 (100 nM) incubated with increasing concentrations of ABT1. The data represent mean values from 3 independent experiments and 9 readings. (**D**) Helicase activity measured with increasing concentrations of ABT1. (**E**) Increasing concentrations of IGHMBP2. (**F**) IGHMBP2 (100 nM) incubated with increasing concentrations of ABT1. The data represent mean values from 3 independent experiments and 9 readings. (**G**) Lanes 1–4 show unwinding analyses of TP31-18mer incubated with 100 nM IGHMBP2 after 1, 3, 5 and 7 minutes, respectively. Lanes 5–8 show unwinding analysis of TP31-18mer incubated with 100 nM IGHMBP2 + 100 nM ABT1 after 1, 3, 5, and 7 minutes, respectively. Lane 9 shows heat-induced TP31-18mer DNA separation (95°C for 5 seconds) used as a positive control. Lane 10 shows Cy3 labeled DNA TP31-18mer alone (negative control). (**H**) Lanes 1–4 show unwinding analysis of TP31-18mer incubated with 100 nM IGHMBP2 after 1, 3, 5, and 7 minutes, respectively. Lane 5 shows unwinding analysis of TP31-18mer incubated with 100 nM IGHMBP2 + 20 nM ABT1 with no incubation. Lanes 6–10 show unwinding analysis of TP31-18mer with 100 nM IGHMBP2 + increasing concentrations of ABT1 (20, 40, 50, 75, 100 nM) after 7 minutes of incubation. (**I**) Quantification of 3 independent experiments of unwinding analyses as shown in **G**. (**J**) Quantification of 3 independent experiments of unwinding analyses as shown in **H**. Arrow indicates the double-stranded duplex, and the arrowhead indicates the resolved duplex. DNA substrate was used at a final concentration of 10 nM. iM, free phosphate produced in μM; RFU, relative fluorescence units. Data are represented as mean ± SD; 1-tailed paired *t* test was used to calculate statistical significance.

Guenther et al. (23) demonstrated that recombinant IGHMBP2 protein functions as a 5′ to 3′ DNA/RNA helicase. To determine whether the association of ABT1 with IGHMBP2 altered IGHMBP2 biochemical activity, the ATPase activity of purified IGHMBP2 alone or in the presence of increasing concentrations (0–120 nM) of purified ABT1 was analyzed. ATPase activity was determined by the amount of free phosphate released as a result of ATP hydrolysis due to enzymatic activity. ABT1 alone demonstrated no intrinsic ATPase activity, even at the highest ABT1 concentration (Figure 4A). IGHMBP2 alone had modest ATPase activity with increased IGHMBP2 concentrations from 80 to 120 nM (Figure 4B). When IGHMBP2 was incubated with increasing concentrations of ABT1 to 120 nM, the ATPase activity of IGHMBP2 was significantly increased 1.96-fold, reaching a plateau when ABT1 reached 120 nM (*P* < 0.0001) (Figure 4C). These studies demonstrate that the association of ABT1 with IGHMBP2 significantly increased the ATPase activity of IGHMBP2.

To determine whether the association of ABT1 with IGHMBP2 alters the helicase activity of IGHMBP2, helicase assays were performed. Increasing concentrations of purified ABT1 up to 120 nM did not demonstrate helicase activity (Figure 4D). IGHMBP2 showed modest helicase activity when monitored against increasing concentrations of IGHMBP2 (Figure 4E). When IGHMBP2 was incubated with increasing concentrations of ABT1 to 120 nM, the helicase activity of IGHMBP2 was significantly increased by 1.41-fold (*P* < 0.00004) (Figure 4F). Interestingly, the helicase activity of IGHMBP2 did not appear to plateau when ABT1 is at 120 nM (Figure 4F). These studies illustrate that the binding of ABT1 to IGHMBP2 significantly increased the helicase activity of IGHMBP2.

IGHMBP2 nucleic acid unwinding rate/processivity was analyzed using a Cy3-labeled DNA substrate. Incubation of the DNA substrate with IGHMBP2 alone for 1, 3, 5, or 7 minutes did not result in significant DNA duplex resolution. Double-stranded template was the primary product, along with partially unwound intermediate (Figure 4, G and I). When IGHMBP2 and ABT1 were incubated together with the DNA substrate for 1, 3, 5, or 7 minutes, unwinding of the duplex occurred with significant duplex resolution at 7 minutes (Figure 4, G and I). When the nucleic acid unwinding rate was determined, the rate for IGHMBP2 was 1.141/min; however, when IGHMBP2 + ABT1 was analyzed, the rate increased significantly to 5.958/min, a 1.42-fold increase (*P* < 0.001). To determine whether IGHMBP2 processivity was affected by ABT1 concentrations, IGHMBP2 was incubated with increasing ABT1 concentrations from 20 to 100 nM for 7 minutes. The most significant duplex resolution occurred when IGHMBP2 was incubated with 100 nM ABT1 (Figure 4, H and J). When the rate of DNA substrate unwinding/nM protein was calculated for IGHMBP2 alone, it was 0.005/min; however, when ABT1 was added to IGHMBP2, there was a significant increase in the rate of unwinding (0.01/min), with an increased activity of 1.84-fold (*P* < 0.00004). These results demonstrate that ABT1 association with IGHMBP2 increased the processivity of IGHMBP2.

### The IGHMBP2/ABT1 complex associates with the 47S pre-rRNA 5′ ETS.

Esf2, the *S*. *cerevisiae* homolog of ABT1, stably associates with the 35S pre-rRNA 5′ external transcribed spacer and the C+D box U3 snoRNA (35). Esf2 depletion inhibits small subunit (SSU) processome assembly, early pre-rRNA processing, and U3 snoRNA release from the 35S pre-rRNA (35). siRNA reduction of ABT1 resulted in accumulation of 47S, 34S, and 30S pre-rRNA intermediates (37).

To determine whether the purified ABT1/IGHMBP2 complex associates with the 47S pre-rRNA 5′ ETS and/or U3 snoRNA, human U3 snoRNA and 204 nucleotides of the human 5′ ETS, from position 414 (site of internal processing) to position 618, were in vitro transcribed. This nucleotide segment of the 5′ ETS is highly conserved between mouse and human in otherwise divergent sequences. A scrambled in vitro transcribed RNA of the same size was used as a control.

**Figure 5:**
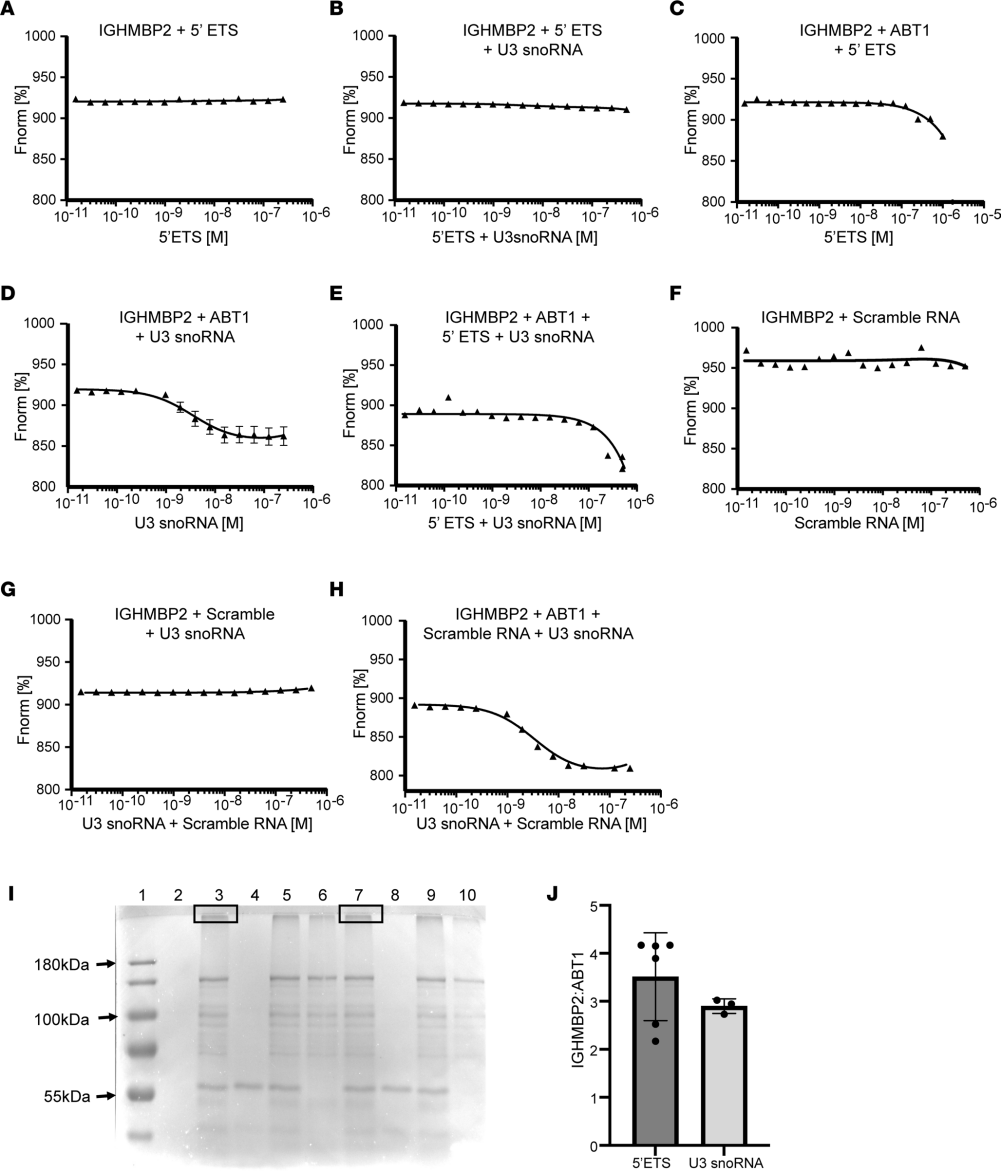
IGHMBP2 and ABT1 associate with the 5′ external transcribed spacer and U3 snoRNA. Binding isotherms with normalized fluorescence (Fnorm[%]) plotted. IGHMBP2 is RED-Tris-NTA labeled. The 5′ ETS + U3 snoRNA and scramble RNA + U3 snoRNA were annealed. Single RNAs were analyzed with starting concentrations of 1 μM, and 2 RNAs were analyzed with starting concentrations of 500 nM each. (**A**) 100 nM IGHMBP2 + 5′ ETS RNA. (**B**) 100 nM IGHMBP2 + 5′ ETS RNA + U3 snoRNA. (**C**) 100 nM IGHMBP2 + 100 nM ABT1 + 5′ ETS RNA; *K_D_* ≥ 697 nM. (**D**) 100 nM IGHMBP2 + 100 nM ABT1 + U3 snoRNA; *K_D_* = 35 nM. (**E**) 100 nM IGHMBP2 + 100 nM ABT1 + 5′ ETS RNA + U3 snoRNA; *K_D_* = 28 nM. (**F**) 100 nM IGHMBP2 + scramble RNA. (**G**) 100 nM IGHMBP2 + scrambled RNA + U3 snoRNA. (**H**) 100 nM IGHMBP2 + 100 nM ABT1 + scramble RNA + U3 snoRNA; *K_D_* = 1 nM. Each panel is taken from 9 readings of 3 independent experiments; data are shown as mean ± SD. Values are plotted using MO.Affinity Analysis software, and the data were fit to a quadratic equation using nonlinear regression. The data are fit to a quadratic equation to determine the *K_D_*. (**I**) SDS PAGE gel of UV crosslinked products. Samples were crosslinked for 60 minutes unless stated. Lane 1 shows protein marker; lane 2 shows 5′ ETS RNA; lane 3 shows IGHMBP2, ABT1, 5′ ETS RNA; lane 4 shows ABT1, 5′ ETS RNA; lane 5 shows IGHMBP2, ABT1, 5′ ETS RNA crosslinked 30 minutes; lane 6 shows IGHMBP2, 5′ ETS RNA; lane 7 shows IGHMBP2, ABT1, U3 snoRNA; lane 8 shows ABT1, U3 snoRNA; lane 9 shows IGHMBP2, ABT1, U3 snoRNA crosslinked 30 minutes; and lane 10 shows IGHMBP2, U3 snoRNA. Boxed area in lanes 3 and 7 represent the samples submitted for mass spectrometry. (**J**) Mass spectrometry IGHMBP2/ABT1 ratio with the 5′ ETS or U3 snoRNA. Three biological samples were normalized to total intensity to quantify IGHMBP2 and ABT1 ± SD.

When IGHMBP2 was incubated with the 5′ ETS RNA or annealed 5′ ETS RNA and U3 snoRNA, and analyzed by MST, the binding reactions were considered unstable (Figure 5, A and B). When the IGHMBP2 was incubated with ABT1 and the 5′ ETS, the binding affinity was > 697 nM, suggesting that there was a weak, unstable association (Figure 5C); however, when ABT1 was incubated with IGHMBP2 and U3 snoRNA, the binding affinity increased substantially to 35 nM, suggesting that ABT1 stabilized the binding interaction between IGHMBP2 and U3 snoRNA (Figure 5D). Interestingly, when annealed 5′ ETS + U3 snoRNA were incubated with IGHMBP2 and ABT1, the *K_D_* was 28 nM, suggesting that ABT1 binding induced a conformational change in IGHMBP2 that exposed an RNA binding site (Figure 5E). Little to no change in the thermophoresis was detected when IGHMBP2 was incubated with the scramble RNA alone or when IGHMBP2 was incubated with the scramble RNA and U3 snoRNA, suggesting that these were unstable interactions (Figure 5, F and G). However, when IGHMBP2 was incubated with ABT1, U3 snoRNA, and the scramble RNA and analyzed by MST, the binding affinity was very strong (*K_D_* = 1 nM).These results suggest that ABT1 binding to IGHMBP2 induced a conformational change in IGHMBP2, exposing a RNA binding site, which in turn bound the scramble RNA with very high affinity (Figure 5H).

When IGHMBP2 was incubated with ABT1 and 5′ ETS RNA or U3 snoRNA and was crosslinked, upshifted products were detected (Figure 5I). Western blot analyses using ABT1 and IGHMBP2 antibodies detected both proteins in these crosslinked samples (our unpublished observations). Negative controls did not demonstrate this upshifted product (Figure 5I).

The bands associated with the upshift products IGHMBP2/ABT1/5′ ETS RNA and IGHMBP2/ABT1/U3 snoRNA were isolated and sent for mass spectrometry. Mass spectrometry demonstrated that ABT1 and IGHMBP2 were present in each sample, and the ratio of IGHMBP2/ABT1 bound to the 5′ ETS or U3 snoRNA was nearly equivalent (Figure 5J). These results demonstrate that the binding of ABT1 to IGHMBP2 induced a conformational change within IGHMBP2. This conformational change enhanced the IGHMBP2/ABT1 complex association with the 5′ ETS and U3 snoRNA and suggests that this complex functions in pre-rRNA processing.

### FVB-Ighmbp2^nmd/nmd^ mice.

The in vitro studies above demonstrate that the association of ABT1 with IGHMBP2 increased the ATPase and helicase activity of IGHMBP2 — functions that are likely important disease-associated activities. To determine whether the interaction of ABT1 with IGHMBP2 altered disease pathology, FVB-*Ighmbp2^nmd/nmd^* mice were used, as they had less variable lifespan.

SMARD1 is a result of recessive inheritance of mutant *IGHMBP2* alleles. FVB-*Ighmbp2^+/nmd^* mice were crossed, and pups were genotyped using primers that amplify a 458 bp fragment. Amplicons were restriction enzyme digested with *Dde*I, a unique restriction site located only within the mutant allele. WT mice were identified by a 450 bp fragment and an 8 bp fragment (not resolved) following gel electrophoresis (Supplemental Figure 2). Heterozygous (*Ighmbp2^+/nmd^*) mice were identified by the presence of the WT 450 and 8 bp bands as well as 268 and 182 bp products representing the mutant allele (Supplemental Figure 2). Homozygous (*Ighmbp2^nmd/nmd^*) animals were identified by the exclusive presence of the 268 bp and 182 bp products (Supplemental Figure 2). As controls, an undigested WT sample and a no-template control are shown. For these studies, FVB-*Ighmbp2^+/nmd^* mice were used as breeders and are referred to as *Ighmbp2^nmd^* or FVB-*nmd*.

### AAbt1 modifies SMARD1-nmd disease pathology.

**Figure 6:**
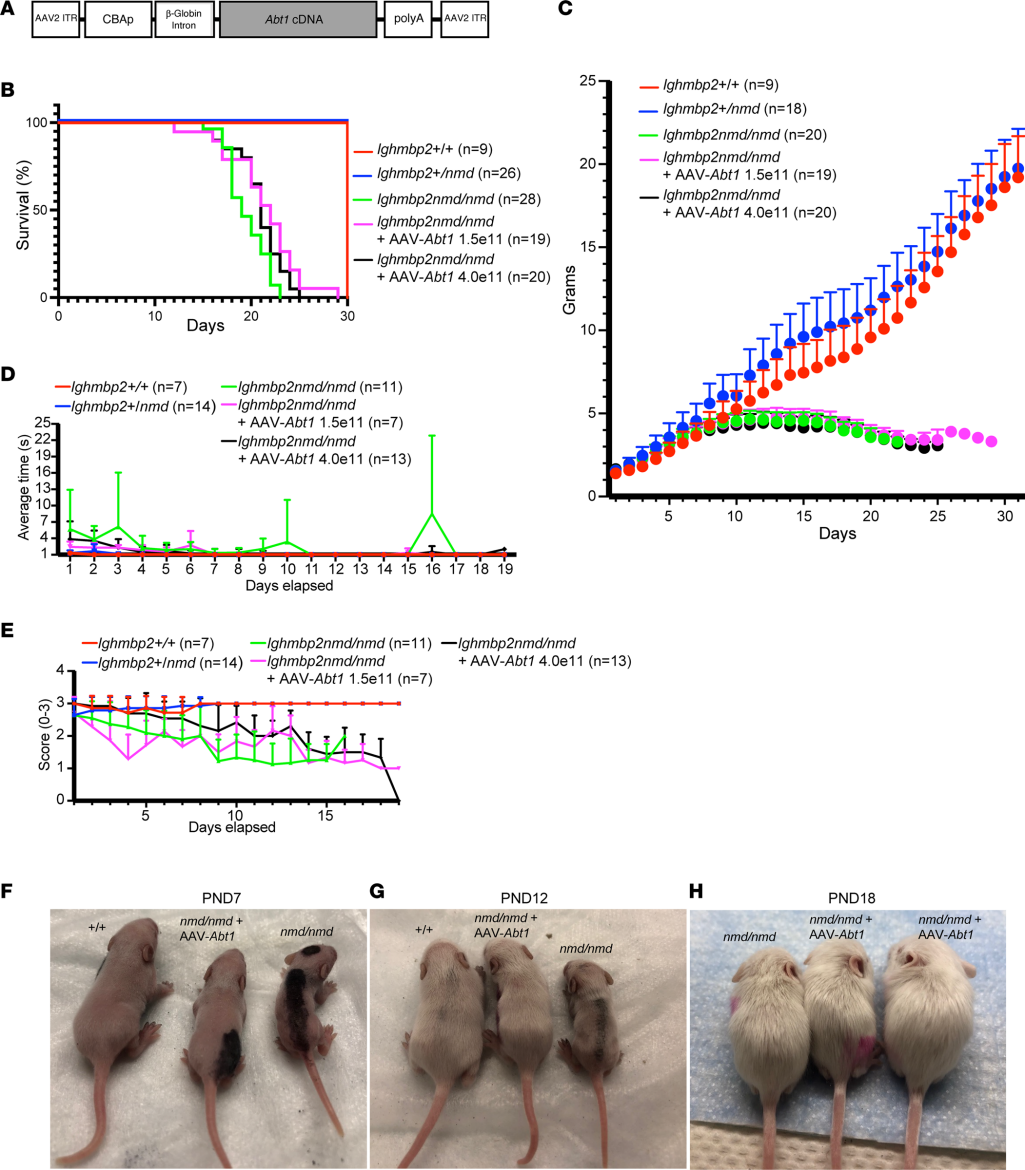
scAAV9-*Abt1* delivery modifies the *Ighmbp2^nmd/nmd^* phenotype. (**A**) Illustration of the self-complementary AAV9-*Abt1* vector. The chicken β-actin promoter is used to express the *Abt1* cDNA along with an optimized intron within the 5′ leader sequence and a synthetic poly A site. (**B**–**E**) WT (+/+), *Ighmbp2^nmd/nmd^*, and *Ighmbp2^nmd/nmd^* mice i.c.v. injected with scAAV9-*Abt1* (1.5 ***×*** 10^11^ or 4 ***×*** 10^11^ viral genomes) are evaluated for lifespan (**B**), weight (**C**), time-to-right (P7–P26) (**D**), and hindlimb splay (P7–P26) (**E**). (**F**–**H**) Representative images of WT (+/+), *Ighmbp2^nmd/nmd^*, and *Ighmbp2^nmd/nmd^* mice injected with scAAV9-*Abt1* (4 ***×***10^11^ viral genomes) at P7 (**F**), P12 (**G**), and P18 (**H**). Statistical analyses for lifespan and weight was 2-way ANOVA with Tukey’s multiple-comparison test; data are shown as mean ± SEM. Statistical analyses for TTR and HLS was ordinary 1-way ANOVA with Tukey’s multiple-comparison test.

To determine whether *Abt1* could improve hallmarks of SMARD1 disease, lifespan, weight, TTR, and HLS were evaluated in *Ighmbp2*^+/+^, *Ighmbp2*^+/–^, *Ighmbp2^nmd/nmd^*, and *Ighmbp2^nmd/nmd^* animals i.c.v. injected with scAAV9-*Abt1* (Figure 6).

We tested motor function starting at P7 using TTR and HLS. For the TTR assay, the time it took for an animal placed on its back to right itself onto all 4 paws was recorded in seconds from P7 to P25. In WT mice, the animal righted itself immediately. For the HLS assay, the mouse was suspended at the base of the tail, and the extension of the HLS was scored from 3 to 0 from P7 to P25; a full hindlimb extension was scored as a 3, a partial extension in line with the body was scored as a 2, a weak extension slightly past midline was scored as a 1, and the absence of hindlimb extension was scored as a 0. In WT mice, there was full hindlimb extension.

To determine whether *Abt1* could improve SMARD1 disease pathology, scAAV9-*Abt1* virus was i.c.v. injected into FVB-*nmd* mutant mice at P2, and lifespan, weight, TTR, and HLS were evaluated (Figure 6, A–E). Survival analyses demonstrate a median survival for *Ighmbp2^nmd/nmd^* mice of 19 days, while mutant mice injected with scAAV9-*Abt1* at 1.5 ***×*** 10^11^ viral genomes had a median survival of 22 days, and mutant mice injected with scAAV9-*Abt1* at 4 ***×*** 10^11^ viral genomes survived to 21 days. There was a statistical difference between *Ighmbp2^nmd/nmd^* mice and *Ighmbp2^nmd/nmd^* + scAAV9-*Abt1* (1.5 ***×*** 10^11^) mice (*P* < 0.0064) as well as between *Ighmbp2^nmd/nmd^* mice and *Ighmbp2^nmd/nmd^* + scAAV9-*Abt1* (4 ***×*** 10^11^) mice (*P* < 0.036). At 20 days, 63% of mutant mice injected with scAAV9-*Abt1* (1.5 ***×*** 10^11^) were alive, as compared with 36% of uninjected *Ighmbp2^nmd/nmd^* mice. These differences become more pronounced at 23 days, where 26% of mutant mice injected with scAAV9-*Abt1* (1.5 ***×*** 10^11^) were alive as compared with 0% of uninjected *Ighmbp2^nmd/nmd^* mice. When littermates were observed at P1, the weight of pups — WT or mutant — was roughly the same. *Ighmbp2^nmd/nmd^* mice began to show deficits in growth and weight by P8 that became more pronounced as disease progresses (Figure 6, B, C, and F–H). By P12, scAAV9-*Abt1*–injected mutant mice showed a noticeable difference in weight and size as compared with noninjected mutant mice; however, scAAV9-*Abt1*–injected mutant mice never reached WT size or weight (Figure 6, C and F–H).

TTR was assessed from P7 to P25, and *Ighmbp2^nmd/nmd^* mice had an increased TTR over WT mice (*P* < 0.0004) (Figure 6D). TTR was significantly improved in *Ighmbp2^nmd/nmd^* mice following scAAV9-*Abt1* injection (1.5 ***×*** 10^11^, *P* < 0.0152; 4 ***×*** 10^11^, *P* < 0.0471). When HLS was measured from P7 to P25, there was a significant difference between WT and *Ighmbp2^nmd/nmd^* mice (*P* < 0.0001) (Figure 6E). *Ighmbp2^nmd/nmd^* mice developed hindlimb weakness early that progressed with disease, as observed with diminished HLS and retrenched hindlimbs. Throughout the 19 days of assessment, *Ighmbp2^nmd/nmd^* + scAAV9-*Abt1* (4 ***×*** 10^11^) animals trended to have better HLS then *Ighmbp2^nmd/nmd^* animals, but because of the variation within each group, the deviation did not reach statistical significance.

### Improvement of the Ighmbp2^nmd/nmd^ phenotype is not a consequence of altered Ighmbp2 splicing.

**Figure 7:**
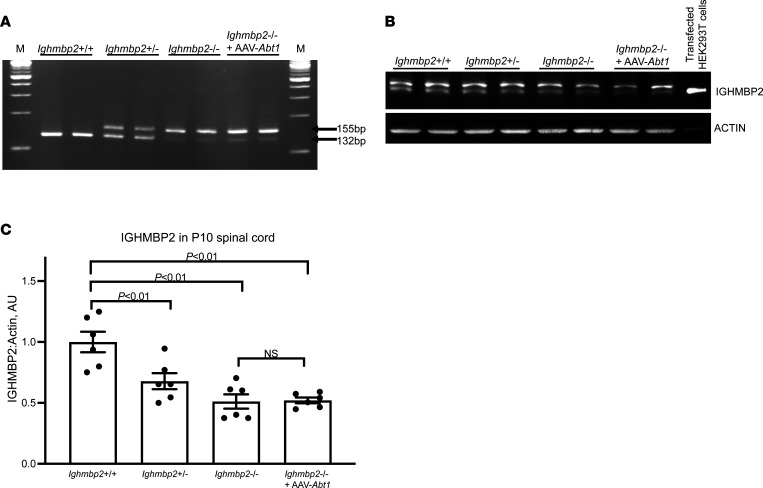
scAAV9-*Abt1* delivery in *Ighmbp2^nmd/nmd^* mice does not modify *Ighmbp2* splicing nor IGHMBP2 protein levels. WT (+/+), *Ighmbp2*^+/nmd^*, Ighmbp2^nmd/nmd^*, and *Ighmbp2^nmd/nmd^* mice injected with scAAV9-*Abt1* (4 ***×*** 10^11^ viral genomes) are evaluated. (**A**) Representative RT-PCR of P10 spinal cords. M, New England Biolabs 100 bp DNA marker. (**B**) Representative Western blot of P10 spinal cords. HEK293T cells transfected with ssAAV9-*IGHMBP2* served as the IGHMBP2 positive control. Actin served as the loading control. In total, 20 μg of protein was added to each lane. (**C**) Quantification of IGHMBP2/actin ratio based on Western blots of P10 spinal cords. Densitometry data determined by Western blot were analyzed by PROC GLM of Statistical Analysis Systems (v9.4). F test was used to determine treatment effects, and Duncan multiple range test was used for differences between groups; data are shown as mean ± SEM. *n* = 6.

The nmd phenotype is a result of a A-to-G mutation in intron 4 of *Ighmbp2* that creates a cryptic splice donor resulting in the inclusion of an additional 23 nucleotides (28). In mutant tissue extracts, ~20% of *Ighmbp2* mRNAs are WT, while 80% are mutant (28). To determine whether ABT1 modified the FVB-nmd phenotype by altering splicing or improving expression of *Ighmbp2*, reverse transcription PCR (RT-PCR) was performed on P10 spinal cord extracts from *Ighmbp2*^+/+^, *Ighmbp2^+/nmd^*, *Ighmbp2^nmd/nmd^,* and *Ighmbp2^nmd/nmd^* pups i.c.v. injected with scAAV9-*Abt1* (4 ***×*** 10^11^ viral genomes) (Figure 7A). WT spinal cord extracts, as predicted, produced a single 132 bp product 100% of the time, while extracts from *Ighmbp2^+/nmd^* mice generated the WT 132 bp product and the mutant 155 bp product (Figure 7A). In FVB-*nmd* mutant mice, the 155 bp product was the predominate band with a significantly reduced WT 132 bp band. In FVB-*nmd* mutant mice injected with scAAV9-*Abt1*, there was not a significant difference in the mutant/WT ratio as compared with FVB-*nmd* mutants. This demonstrates that ABT1 does not change SMARD1-*nmd* disease pathology by shifting splicing toward the FL transcript (Figure 7A). Splicing differences were not detected in P10 brain tissue extracts (our unpublished observations). These results are consistent with those observed with the modifying BAC-27k3 (24).

### Changes in Ighmbp2^nmd/nmd^ disease pathology are not attributed to increased IGHMBP2 protein levels in scAAV9-Abt1–injected mice.

Since i.c.v. injection of scAAV9-*Abt1* does not change FVB-nmd disease pathology by altering *Ighmbp2* splicing, we investigated whether IGHMBP2 protein levels were enhanced in *Ighmbp2^nmd/nmd^* mice following scAAV9-*Abt1* injection. P10 spinal cord extracts from *Ighmbp2*^+/+^, *Ighmbp2^+/nmd^*, *Ighmbp2^nmd/nmd^,* and *Ighmbp2^nmd/nmd^* pups i.c.v. injected with scAAV9-*Abt1* (4 ***×*** 10^11^ viral genomes) were harvested an analyzed by Western blot (Figure 7B). As compared WT tissue extracts, there was a progressive reduction of FL-IGHMBP2 in *Ighmbp2^+/nmd^* and *Ighmbp2^nmd/nmd^* extracts (Figure 7, B and C). P2 injection of scAAV9-*Abt1* did not significantly alter FL-IGHMBP2 levels, suggesting that SMARD1-*nmd* disease pathology was not modified by ABT1 through increasing IGHMBP2 protein (Figure 7, B and C). Protein differences were not detected in P10 brain extracts (our unpublished observations).

### Reduction of IGHMBP2 protein alters ABT1 protein levels in Ighmbp2^nmd/nmd^ mice.

To determine whether reduction of IGHMBP2 in FVB-*nmd* mutants regulated expression of ABT1, P10 spinal cord extracts from *Ighmbp2*^+/+^, *Ighmbp2^+/nmd^*, *Ighmbp2^nmd/nmd^*, and *Ighmbp2^nmd/nmd^* pups i.c.v. injected with scAAV9-*Abt1* (4 ***×*** 10^11^ viral genomes) were examined (Supplemental Figure 3). Reduction of IGHMBP2 changed expression of ABT1 between WT and mutant tissue extracts. scAAV9-*Abt1* injection increased ABT1 levels to essentially WT. These studies demonstrate that the scAAV9-*Abt1* construct was being expressed, and while ABT1 levels were reduced in *nmd* mutants, ABT1 levels were restored to WT following scAAV9-*Abt1* injection (Supplemental Figure 3).

### scAAV9-Abt1 delivery reduces NMJ denervation but does not change muscle fiber size in Ighmbp2^nmd/nmd^ mice.

**Figure 8:**
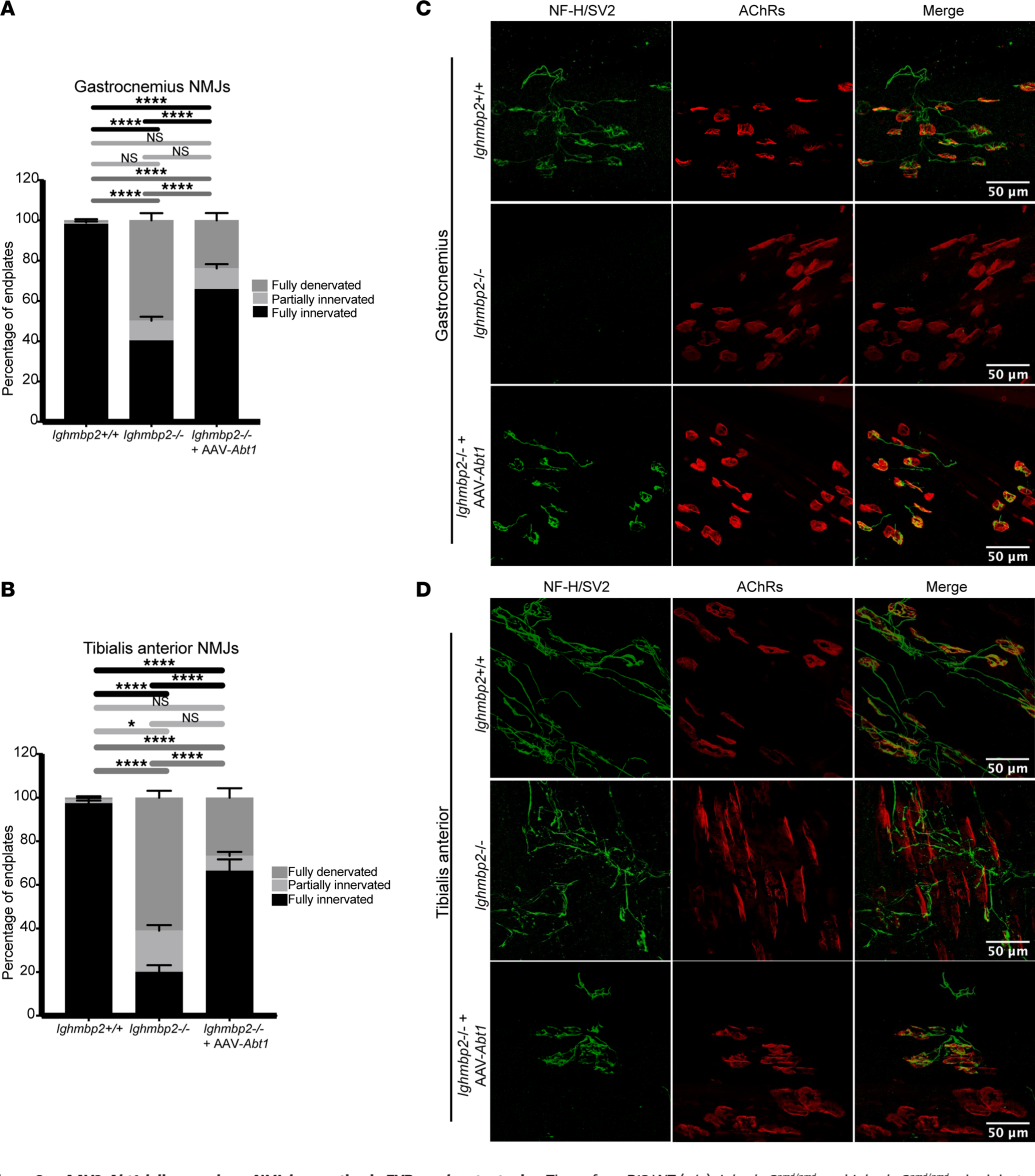
scAAV9-*Abt1* delivery reduces NMJ denervation in FVB-*nmd* mutant mice. Tissue from P12 WT (+/+), *Ighmbp2^nmd/nmd^*, and *Ighmbp2^nmd/nmd^* mice injected with scAAV9-*Abt1* (4 ***×*** 10^11^ viral genomes) was evaluated. (**A**) Quantification of the percentage of end plates of gastrocnemius neuromuscular junctions (NMJs). Assessment of fully denervated (dark gray), partially innervated (light gray), or fully innervated (black) end plates. Statistical significance was determined by 2-way ANOVA with a Tukey’s multiple-comparison post hoc test; ****P *≤* 0.0001. Greater than 100 end plates per cohort were counted and averaged. Data points on graphs represent the average per animal with statistical analysis comparing the average of each animal. (**B**) Quantification of the percentage of end plates of tibialis anterior NMJs. Assessment of fully denervated (dark gray), partially innervated (light gray), or fully innervated (black) end plates. Statistical significance was determined by 2-way ANOVA with a Tukey’s multiple-comparison post hoc test; *P *≤* 0.0331, ****P *≤* 0.0001. Greater than 100 end plates per cohort were counted and averaged. Data points on graphs represent the average per animal with statistical analysis comparing the average of each animal. (**C**) Microscopic images of gastrocnemius tissue sections. (**D**) Microscopic images of tibialis anterior tissue sections. Tissue was assessed by neurofilament heavy chain (NF-H) and synaptic vesicle type 2 (SV2) to label axons and synaptic terminals. bungarotoxin labeled acetylcholine receptors (AChRs). Original magnification, 40***×***. Analyses were performed with GraphPad Prism software. Data are shown as mean ± SEM. Data points on graph represent the average per animal with statistical analysis comparing the average of each animal. Scale bars: 50 μm.

In many neuromuscular diseases, NMJ pathology precedes deficits in motor function. To determine whether scAAV9-*Abt1* delivery could prevent NMJ denervation in FVB-*nmd* mutant mice, P12 gastrocnemius and tibialis anterior were examined. These muscles were shown to be highly susceptible to NMJ denervation in B6-*nmd*^2J^ mutant mice (31). Significant NMJ denervation was observed in FVB-*nmd* mutant mice as compared with WT controls in the gastrocnemius and tibialis anterior (Figure 8). When *Ighmbp2^nmd/nmd^* pups were i.c.v. injected with scAAV9-*Abt1* (4 ***×*** 10^11^ viral genomes), fully denervated NMJs were significantly reduced, although not restored to WT numbers (Figure 8). There was not a significant difference in partially innervated NMJs between WT, mutant, or mutants injected with scAAV9-*Abt1*, suggesting that the NMJ population was either innervated or denervated (Figure 8). Interestingly, while there was a significant difference in the muscle fiber area and perimeter between WT and FVB-*nmd* mutants, scAAV9-*Abt1* delivery did not impact either muscle fiber area or perimeter (Supplemental Figure 4, A–C).

## Discussion

This manuscript identifies ABT1 as the first modifier of SMARD1 disease pathology and provides the first functional mechanism for the IGHMBP2/ABT1 complex in pre-rRNA processing, to our knowledge. These studies also demonstrate that the rescuing activity of BAC-27k3 in B6-*nmd*^2J^ mice may be attributed to ABT1.

Changes in disease pathology in FVB-*nmd* mutants following scAAV9-*Abt1* delivery does not appear to be due to changes in *IGHMBP2* RNA levels or splicing, nor in IGHMBP2 protein levels. These results are consistent with those observed with the rescuing activity of BAC-27k3 (24). These results suggest that local overexpression of ABT1 in *nmd* mutant mice alters the biochemical properties of the residual IGHMBP2, making more IGHMBP2 biochemically active and/or more efficient. However, it cannot be excluded that some changes in disease pathology following scAAV9-*Abt1* delivery might be due to IGHMBP2 independent mechanisms. ABT1 protein levels are reduced in spinal cords of P10 *nmd* mice, suggesting that IGHMBP2-dependent mechanisms might alter ABT1 expression. This is consistent with the proposed mechanism of IGHMBP2 function; low IGHMBP2 results in deficits in pre-rRNA processing that, in turn, effects the fidelity of translation. These studies are similar to those in which low levels of survival motor neuron (SMN) result in reduced ZPR1, a modifier of SMN (40).

scAAV9-*Abt1* delivery significantly reduced NMJ denervation in FVB-*nmd* mutant mice, even within some of the most vulnerable muscles, the gastrocnemius and tibialis anterior. scAAV9-*Abt1* delivery to FVB-*nmd* mutant mice did not alter muscle fiber diameter nor perimeter in these same muscles. One possible explanation could be that reduced IGHMBP2 levels in FVB-*nmd* mice resulted in reduced NMJ innervation, muscle atrophy, and muscle fiber size in *nmd* mutants. scAAV-*Abt1* delivery improved NMJ innervation, but because innervation is required to stimulate muscle fiber regeneration, increases in muscle fiber size were not accomplished during the period from scAAV-*Abt1* expression to when tissue was harvested at P12. Improvements in motor function were likely a result of scAAV-*Abt1* delivery improving the function of existing NMJ-muscle units.

So why do *nmd* mutants die if i.c.v. scAAV9-*Abt1* delivery positively impacts lifespan and clearly reduces NMJ denervation? First, scAAV9-*Abt1* delivery is not gene replacement and IGHMBP2 levels are still significantly reduced in *nmd* mutants. This means that there not only is less IGHMBP2 available, but also less IGHMBP2 for ABT1 to bind and activate. Second, it has been demonstrated for many neuromuscular diseases, including spinal muscular atrophy (SMA), SMARD1, and ALS, that the CNS — in particular, motor neurons — are highly sensitive to the changes brought on by these diseases. It is likely that scAAV9-*Abt1* delivery directly to the CNS at P2 impacts local motor neurons and, therefore, improves some motor function deficits. However, SMARD1 — just like SMA and ALS — is not exclusively a motor neuron disease; therefore, IGHMBP2 deficits in other tissues not reached by scAAV9-*Abt1* delivery remain impacted. Third, it is likely that SMARD1 defects are present prior to expression of scAAV9-*Abt1*; therefore, modest phenotypic changes are observed. While scAAV9-*Abt1* delivery to FVB-*nmd* mutant mice moderately expanded lifespan and some but not all aspects of SMARD1 disease pathology, these results are consistent with modifiers of SMA (41–45).

To understand how scAAV-*Abt1* delivery could be improving SMARD1 disease pathology, we undertook a series of experiments that were based on previous observations that ABT1 coimmunoprecipitated with IGHMBP2 (24). In these studies, we demonstrated that the association of IGHMBP2 with ABT1 was direct and that ABT1 bound IGHMBP2 with high affinity. The binding of ABT1 appeared to induce a conformational change within IGHMBP2; the nature of this conformational change and whether changes in protein structure change IGHMBP2 from an “inactive” to an ”active” conformation is being investigated. Binding of ABT1 to IGHMBP2 did significantly increase the biochemical activity of IGHMBP2 ATPase and helicase activity and processivity. This suggests that ABT1 binding either changes IGHMBP2 conformation activating IGHMBP2 or that the presence of ABT1 regulates IGHMBP2 activity. This is consistent with the association of Esf2, the yeast ABT1 homolog, that binds to the DexD/H box RNA helicase Dbp8 and stimulates Dbp8 helicase activity (36). Additionally, ZPR1 was recently shown to associate with Senataxin, another UPF1-like helicase, where ZPR1 is proposed to regulate Senataxin helicase activity (46).

Previous studies show that Esf2 associates with the 35S pre-rRNA 5′ ETS and is required for pre-rRNA processing (35). Additionally, when ABT1 is reduced in HeLa cells, there is an accumulation of 47S pre-rRNA processing intermediates (37). Here, we demonstrate that IGHMBP2 associated with ABT1 and this complex bound the 47S pre-rRNA 5′ ETS and U3 snoRNA. To our knowledge, our studies presented here are the first to provide a functional mechanism for the interaction between IGHMBP2 and ABT1 and provide insight into SMARD1 disease pathology. Interestingly, a patient with SMARD1 clinical symptoms, but without an IGHMBP2 mutation, was identified with a mutation in Las1L (47). Mutations in Las1L result in pre-rRNA professing defects in the internal transcribed spacer 2 and accumulation of the 32S pre-rRNA intermediate (48). Based on our findings, IGHMBP2 may join a list of proteins that, when mutated, result in pre-rRNA processing defects that lead to neurodegeneration. It will be important to demonstrate whether mutants that abrogate the binding of ABT1 to IGHMBP2 result in reduced IGHMBP2 biochemical activity, association with the 5′ ETS and U3 snoRNA, and pre-rRNA processing defects.

## Methods

### Protein expression and purification.

Human FL-*IGHMBP2* and mouse FL-*Abt1* cDNAs were synthesized (GenScript). The human *IGHMBP2* cDNA was cloned into the pET32b vector (Novagen). Mouse *Abt1* was cloned into the pGEX6P1 vector (Sigma-Aldrich). Mouse ABT1 was chosen, as we also utilized this construct for viral production of scAAV-*Abt1*. BL21 (C2527, NEB) and Rosetta (71401, Sigma-Aldrich) strains were used in the expression assays. pGEX6P1-ABT1 lysate was mixed with GST-beads (70541, Novagen) that were preequilibrated (20 mM Tris [pH 7.5] with 0.1% Triton X-100) with constant rotation at 4°C for 4 hours. Collected beads were washed 4 times (20 mM Tris [pH 7.5] with 0.1% Triton X-100), followed by a single detergent-free wash (50 mM Trish [pH 8.0]). After each wash, beads were centrifuged at 35*g* for 1 minute at 4°C. GST-tagged proteins were eluted with 50 mM Tris (pH 8.0) containing freshly added 20 mM reduced glutathione. His fusion protein production and purification was performed as above using His beads (69670, Novagen). His-bind resin was washed with 3 volumes of the sterile dH_2_O to remove ethanol and washed with 5 volumes of 1***×*** His-tag charge buffer (50 mM NiSO_4_). The resin was allowed to settle by gravity. To remove the charge buffer, resin was washed 3***×*** volumes with 1***×*** His-tag binding buffer (50 mM Imidazole, 0.5M NaCl, 20 mM Tris [pH 7.9]). The clarified lysate was mixed with preequilibrated His beads with constant rotation at 4°C for 4 hours. The settled resin was washed with 3***×*** volume of 1***×*** His-tagged washing buffer (60 mM Imidazole, 0.5M NaCl, 20 mM Tris [pH 7.9]). After each washing, the resin was exposed to 3***×*** volumes with 1***×*** His-tag binding buffer (50 mM Imidazole, 0.5M NaCl, 20 mM Tris [pH 7.9]). The His-tagged protein bound to resin was boiled to 68°C for 10 minutes to remove the protein from resin or a 1***×*** His-tagged elution buffer was used (1.0M Imidazole, 0.5M NaCl, 20 mM Tris [pH 7.9]).

Absolute protein concentration was determined and normalized using a BSA standard curve. Proteins were electrophoresed on SDS-PAGE using either 10% or 12% resolving gels and transferred onto a methanol pretreated PVDF membrane (MilliporeSigma). The membrane was blocked in a 5% BSA-TBST buffer (5% BSA in Tris-buffered saline with containing 0.01% Tween20). Membranes were probed overnight at 4°C with either anti-GST (1:1,000, AB19256, Abcam), anti-His (1:1,000, AB245114, Abcam), anti-ABT1 (141481AP, Proteintech), or anti-IGHMBP2 (NBP3-05096, Novus Biologicals). Actin (1:1,000, A35853, Sigma-Aldrich) served as internal control. Primary and secondary antibodies were diluted in 5% BSA-TBST. Secondary HRP antibodies used were anti-mouse (AP130P, Sigma-Aldrich) and anti-rabbit (A16023, Thermo Fisher Scientific). An ECL Chemiluminescence Kit (34078, Thermo Fisher Scientific) and ChemiDoc Imaging System (Bio-Rad) were used to detect protein bands.

For immunoprecipitation, 4 mg of protein was used. In total, 100 μL A/G beads (20421, Thermo Fisher Scientific) was used for 1 mg/mL bacterial cell lysate. Cell lysates were preincubated with AG beads for 1 hour at 4°C to remove nonspecific proteins. The preclear lysate was incubated overnight at 4°C with primary antibodies. Antibodies were used at 0.5 μg/1 mg protein ratio included anti-GST (19256, Abcam), anti-His (AB245114, Abcam), anti-ABT1 (141481AP, Proteintech), and anti-IGHMBP2 (MABE162, Sigma-Aldrich). At the end of the incubation, samples were centrifuged at 1,000*g* for 2 minutes at 4°C, and supernatant was removed and washed 3 times with wash buffer (10 mM Tris [pH 7.4], 1 mM EGTA, 150 mM NaCl, 1%Triton X-100, with protease inhibitor cocktail). The complex was eluted from the beads by boiling samples in loading buffer with SDS.

### DLS analysis.

Purified protein samples (1 mg/mL) were diluted to 1:10 in PBS. Size and distribution were measured using the Zetasizer (Malvern Panalytical). After instrument calibration, the temperature was set at 24°C for sample measurements. The particle scatter intensity was proportional to the square of the molecular weight.

### MST binding analysis.

MST was performed using tagged proteins labeled with the RED fluorescent dye NT-647. Sample labeling and processing were performed according to manufacturer instructions (MO-LOO8, NanoTemper Technologies). RED-Tris-NTA fluorescence excitation and emission maxima were at 650 nm and 670 nm, respectively. The MST data were analyzed with the MO.Affinity Analysis software (NanoTemper Technologies) and fit to a quadratic equation using nonlinear regression to determine the *K_D_* value.

### ATPase assay.

The colorimetric determination of ATPase activity was performed using a malachite green assay as described (ab272520, Abcam). The reagents and assay buffers were prepared without free phosphate. Phosphate standards were prepared from 0 to 50 μM. In total, 100 nM IGHMBP2 was used, and ABT1 was used at increasing concentrations up to 120 nM. Absorbance was measured at 620 nm to calculate ATPase activity.

### Helicase assay.

HTS1 (5′-GATCTGAGCCTGGGAGCT-3′ Cy3 labeled; IDT) was used as the DNA substrate at 10 nM. In total, 100 nM of purified protein and DNA substrate were incubated at 37°C for 10 minutes. A 10***×*** helicase buffer (500 mM Tris-HCL [pH 7.5], 0.01% BSA, 10 mM DTT, 20 mM MgCl_2_) and 5 mM ATP were added to the preincubated sample and then incubated for an additional 30 minutes at 37°C. The reaction was stopped and monitored for absorbance at 620 nm. For the kinetic measurements, the absorbance was the direct reflection of the fluorescence released by the enzymatic activity of unwinding the DNA substrate.

### Gel mobility shift assay.

The TP31-18mer sequences (5′-CGCAGTCTTCTCCTGGTGCTCGAACAGTGAC-3′, 3′-ACCACGAGCTTGTCACTG-5′*) were synthesized and annealed (IDT; the asterisk refers to the location of the Cy3 label).

Cy3-labeled DNA substrate was used at a concentration of 10 nM. Purified IGHMBP2 (100 nM) and ABT1 (100 nM) proteins were incubated with TP31-18mer and 20 mM HEPES (pH 7.5), 20 mM NaCl, 5 mM MgCl_2_, 1 mM DTT, 0.1 mg/mL BAS, and 5% glycerol for 1, 3, 5 or 7 minutes at 37°C. Samples were run on a 6% nondenaturing polyacrylamide gel and then imaged using a phosphorimager (FLA 5000, FujiFilm).

### RNA binding studies.

In total, 204 nucleotides of the human 5′ ETS starting at position 414 (site of internal processing) to position 618 was synthesized (GenScript) and were then in vitro transcribed according to manufacturer’s instructions (AM1354, Invitrogen). A scrambled sequence of the same size was synthesized and in vitro transcribed as a control. The human U3 snoRNA was synthesized (GenScript) and then in vitro transcribed. Transcribed RNAs were purified using MEGAclear (AM1908, Invitrogen) as per the manufacturer’s instructions. Preincubated RNA-protein reactions were transferred to ice and irradiated in an ultraviolet crosslinker at 254 nm for 120 seconds (GS-gene UV chamber, Bio-Rad). Samples were diluted 1:1 with nuclease-free water and then treated with RNase A at 37°C for 15 minutes. Samples were resolved on a 12% SDS polyacrylamide gel.

MST binding analyses were performed using NT-647–labeled IGHMBP2 according to the manufacturer (MO-L008, NanoTemper Technologies). Protein concentrations were 100 nM for IGHMBP2 and ABT1. Fluorescence excitation and emission maxima were measured at 650 nm and 670 nm, respectively. Thermophoretic data were analyzed with MO affinity analysis and plotted with nonlinear regression.

Transcribed 5′ ETS and U3 snoRNAs were purified and used for protein crosslink. The RNA-Protein crosslinked samples boiled under denaturing conditions and were resolved on a 12% SDS polyacrylamide gel. Gels were transferred onto a PVDF membrane (MilliporeSigma) and blocked with 5% BSA-TBST buffer for 1 hour. Membranes were incubated overnight at 4°C with either anti-ABT1 (141481AP, Proteintech) or anti-IGHMBP2 (NBP3-05096, Novus Biologicals). Primary and secondary antibodies were diluted in 5% BSA-TBST. Rabbit secondary HRP antibodies used included anti-rabbit (A16023, Thermo Fisher Scientific). An ECL Chemiluminescence Kit (34078, Thermo Fisher Scientific) and the ChemiDoc Imaging System (Bio-Rad) were used.

### Mass spectrometry.

Crosslinked samples of IGHMBP2, ABT1, and 5′ ETS or crosslinked samples of IGHMBP2, ABT1, and U3 snoRNA were boiled under denaturing conditions and were resolved on a 12% SDS polyacrylamide gel to separate free RNA and protein from complex. Coomassie stained 1D gel bands were trypsin digested and extracted. Samples were lyophilized and resuspended and LCMS run on a OrbitrapXL. All raw data files were analyzed using FragPipe. Data files were searched against Uniprot-ALL species.

### RT-PCR.

Total RNA was extracted from P10 whole spinal cords of *Ighmbp2*^+/+^, *Ighmbp2*^+/nmd^, *Ighmbp2*^nmd/nmd^*,* or *Ighmbp2^nmd/nmd^* + scAAV9-*Abt1* (4 ***×*** 10^11^ viral genomes) mice using TRIzol reagent (Invitrogen). RNA was reverse transcribed with SuperScript III Reverse Transcriptase (Thermo Fisher Scientific). The cDNA was used as a template for PCR with forward primer 5′-CGCCTGAAAAAAGCACTGATGAC-3′ and reverse primer 5′-TGTGTTGTAGAAAGAGAGTGGGG-3′ (IDT). The cycling conditions for PCR were (a) 93°C for 3 minutes, (b) 34 cycles of 30 seconds at 93°C, 58°C for 30 seconds, and 30 seconds at 72°C, and (c) a final extension for 5 minutes at 72°C. The PCR products were analyzed on 4% agarose electrophoresis gel. The experiments were done in triplicate using independent samples.

### SDS PAGE and Western blotting analysis.

At P10, mice were sacrificed using 2.5% isoflurane. Whole spinal cord tissue samples were immediately flash frozen in liquid nitrogen and stored at –80°C freezer. Protein was extracted with JLB buffer (50 mM Tris [pH 8.0], 150 mM NaCl, 10% glycerol, 20 mM NaH_2_PO_4_, 50 mM NaF, 2 mM EDTA) supplied with complete miniprotease inhibitor cocktail (Roche) and boiled in SDS loading buffer (50 mM TRIS [pH 6.8], 150 mM NaCl, 2% SDS, 20% glycerol, 0.02% bromophenol blue). To achieve reducing conditions, 5% β-mercaptoethanol was added to the samples. In total, 20 μg of total protein was separated by gel electrophoresis using an 8% SDS-PAGE gel for IGHMBP2 protein analysis or 12% SDS-PAGE gel for ABT1. Proteins were transferred to Immobilon P PVDF membrane (MilliporeSigma). The membrane was incubated sequentially with 5% nonfat dry milk for 1 hour at room temperature and with anti-IGHMBP2 (1:1,000, NBP3-05096, Novus Biologicals), anti-ABT1 (1:600, 141481AP, Proteintech), or anti-actin (1:3,000; A3853, Sigma-Aldrich) primary antibodies at 4°C overnight. HRP-conjugated anti-rabbit secondary antibody (A16023, Thermo Fisher Scientific) was used to detect IGHMBP2 and ABT1 and HRP-conjugated anti-mouse antibody (AP130P, Sigma Aldrich) to detect actin. Membranes were reacted with chemiluminescent substrate (34078, Thermo Fisher Scientific). Images were acquired using a BioSpectrum 816 Imaging System (UVP). Densitometry quantification of the band of interest was determined by Image Studio software (V5.2, Li-COR) with a box drawn around the band and background density subtracted. Densitometry data were analyzed by PROC GLM of Statistical Analysis Systems (v9.4). An F test was used to determine treatment effects and Duncan’s multiple range test for differences between groups.

### Animals and genotyping.

The FVB-*Ighmbp2^nmd^* mouse was used for these studies. Animals were extensively backcrossed. Males and females were used in each cohort. Genotyping of neonatal pups was performed at P1. Genomic DNA was obtained using the DNA isolation protocol from the Jackson Laboratory. The FVB-*Ighmbp2^nmd^* line was genotyped using forward primer 5′-CTGGGTGCTTAGGTGATGGT-3′ and reverse primer 5′-CTGGCAGAAGACCCATGATT-3′ primers (IDT) and GoTaq green master mix (M3001, Promega). PCR conditions were 94°C denaturing for 3 minutes, followed by 39 cycles of 94°C denaturing for 30 seconds, 60°C annealing for 30 seconds, and 72°C extension for 1 minute; the final extension was 72°C for 5 minutes. Amplicons were digested with *Dde*I and separated on a 2% gel to differentiate WT from mutant alleles.

### Generation of scAAV9-Abt1 virus and i.c.v. injections.

The self-complementary AAV9-*Abt1* vector was used in these studies (49). Expression of *Abt1* is driven by the ubiquitously expressing chicken β-actin promoter (CBA), along with an optimized intron within the 5′ leader sequence and a synthetic poly A site. The CBA promoter has early, robust, and sustained expression. AAV9 has tropism for many CNS tissues as well as peripheral tissues. AAV9 has the ability to enter neuronal and nonneural cells (astrocytes and microglia) in the CNS (30, 50–53). Viral particles were generated in HEK293T cells (ATCC CRL-3216) and purified using 3 CsCl density-gradient ultracentrifugation steps followed by dialysis against PBS buffer as previously described (49, 54). Number of viral genomes were determined by quantitative PCR (qPCR) using SYBR green (1902522, Thermo Fisher Scientific). Animals were injected via i.c.v. injection at 1.5 ***×*** 10^11^ or ~4 ***×*** 10^11^ viral genomes on P2.

### Motor function tests.

Motor function was evaluated using the TTR assay from P7 to P26. Each pup was placed on its back, and the time it took for the pup to turn over and stabilize on all 4 paws was recorded (51, 55). The maximum attempted time was 30 seconds. Hindlimb function was assessed using HLS, a spontaneous reflex reaction of mice to spread out their hind legs when they are held by the base of the tail (51, 55–58). HLS was initiated at P7–P26. To administer the test, mice were held approximately 0.5 inches from the base of their tail for ~2 seconds. The relative distance between the splayed hindlimbs was scored 0–3. A 3 was recorded for a normal full hindlimb extension, 2 for an extension just in line with the body, 1 for a slight extension and some movement of the hindlimbs, and 0 for no extension/completely retracted hindlimbs.

### Skeletal muscle IHC.

P12 animals were sacrificed and perfused with 4% PFA, followed by a postfixing in 4% PFA for 48 hours at 4°C. Skeletal muscle was dissected and cryoprotected in 30% sucrose overnight. Cryoprotected muscles were embedded in OCT media and cryosectioned at 16 μm with 3–4 continuous cross sections per muscle. Sections were stained with anti-laminin primary antibody (1:200, L9393, Sigma-Aldrich) and donkey anti–rabbit Alexa Fluor 594 secondary antibody (1:400, 711-585-152, Jackson ImmunoResearch). Imaging was obtained using a Leica DM5500 B fluorescence microscope (Leica Microsystem) under 40***×*** magnification with 3–4 images per muscle. Muscle fiber area and perimeter measurements were analyzed by manually tracing myofibers using ImageJ Software (NIH) in a blinded manner.

### NMJ IHC.

Whole-mount preparations were postfixed in 4% PFA following perfusion of each mouse. Anti–neurofilament heavy chain (anti–NF-H) (1:2,000, AB5539, MilliporeSigma) and anti–synaptic vesicle 2 (anti-SV2) (1:200, YE269, Life Technologies) primary antibodies, followed by donkey anti–chicken Alexa Fluor 488 (1:400, 703-545-155, Jackson ImmunoResearch) and goat anti–rabbit Alexa Fluor 488 (1:200, 111-545-003, Jackson ImmunoResearch) secondary antibodies, were used to label the axon and synaptic terminal. Acetylcholine receptors were labeled with Alexa Fluor 594–conjugated α-bungarotoxin (1:200, B13423, Life Technologies). Representative images were obtained using a laser scanning confocal microscope at 40***×*** magnification (Leica TCS SP8, Leica Microsystems Inc). NMJ analyses was performed in a blinded manner on at least 3 randomly selected fields of view per muscle at 40***×*** magnification. Images were analyzed based on the end plate overlap with the synaptic terminal. End plates with missing overlapping terminal were considered fully denervated, end plates with partial overlap were considered partially denervated, and end plates with complete overlap were considered fully innervated.

### Statistics.

All in vitro experiments were performed in at least 3 biological replicates for reproducibility of data. For quantification of unwinding, data are represented as mean ± SD. A 1-tailed paired *t* test was used to calculate statistical significance. *P* < 0.05 was considered as statistically significant. Densitometry data determined by Western blot were analyzed by PROC GLM of Statistical Analysis Systems (v9.4). F test was used to determine treatment effects, and Duncan multiple range test was used for differences between groups. Skeletal muscle fiber statistical significance was determined using 1-way ANOVA with a Tukey’s multiple-comparison post hoc test. NMJs were analyzed by a 2-way ANOVA with a Tukey’s multiple-comparison post hoc test. Analyses were all performed with GraphPad Prism software. Data are shown as mean ± SEM. Data points on graphs represent the average per animal, with statistical analysis comparing the average of each animal.

### Study approval.

All experimental procedures were approved by the University of Missouri’s IACUC and were performed according to the guidelines set forth in the *Guide for the Care and Use of Laboratory Animals* (National Academies Press, 2011).

## Author contributions

GPV conducted experiments, acquired data, analyzed data, and edited the manuscript. MOGK, ZCL, and RPR conducted experiments and acquired data. JM conducted experiments, acquired data, and analyzed data. SMRH conducted experiments, acquired data, analyzed data, and edited the manuscript. SAH conducted experiments. CLL and KS analyzed data and edited the manuscript. MAL designed research studies, conducted experiments, acquired data, analyzed data, prepared the manuscript, and edited the manuscript.

## Supplementary Material

Supplemental data

## Version 1 12/08/2022

In-Press Preview

## Version 2 01/24/2023

Electronic publication
