# Supplementary material for: ABT1 modifies SMARD1 pathology via interactions with IGHMBP2 and stimulation of ATPase and helicase activity
Source: JCI Insight. 2023 Jan 24;8(2):e164608. doi: 10.1172/jci.insight.164608 (PMC9977310; doi:10.1172/jci.insight.164608)
Supplement: Supplemental data [file jciinsight-8-164608-s190.pdf]

Supplemental figures

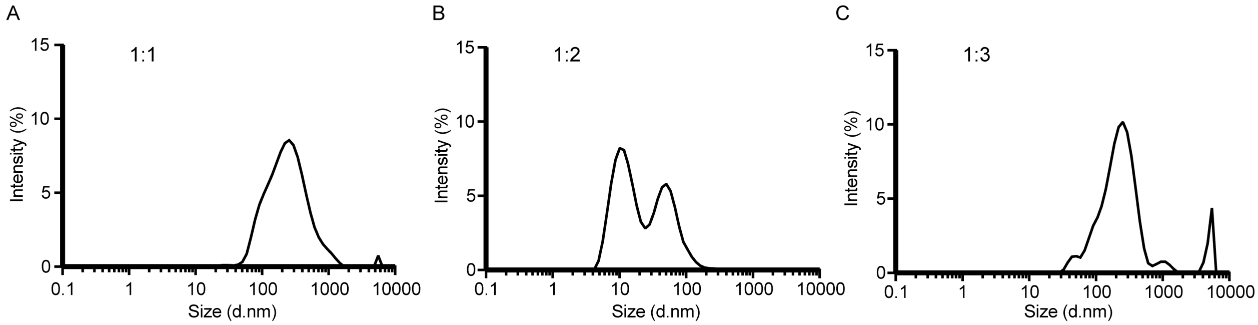

Supplemental Figure 1. The stoichiometry of IGHMBP2:ABT1 is 1:1. Representative graphs depicting the intensity and size from dynamic light scattering (9 readings of three independent experiments). **(A)** DLS reading for 100μM IGHMBP2 and 100μM ABT1, a 1:1 ratio. Peak mean intensity 8.55 with SD 0.47. **(B)** DLS reading for 100μM IGHMBP2 and 200μM ABT1, a 1:2 ratio. Peak mean intensity for peak 1 is 8.21 with SD 1.10. Peak mean intensity for peak 2 is 5.80 with SD 2.11. **(C)** DLS reading for 100μM IGHMBP2 and 300μM ABT1, a 1:3 ratio. Peak mean intensity 10.18 with SD 2.82. μM=micromolar.

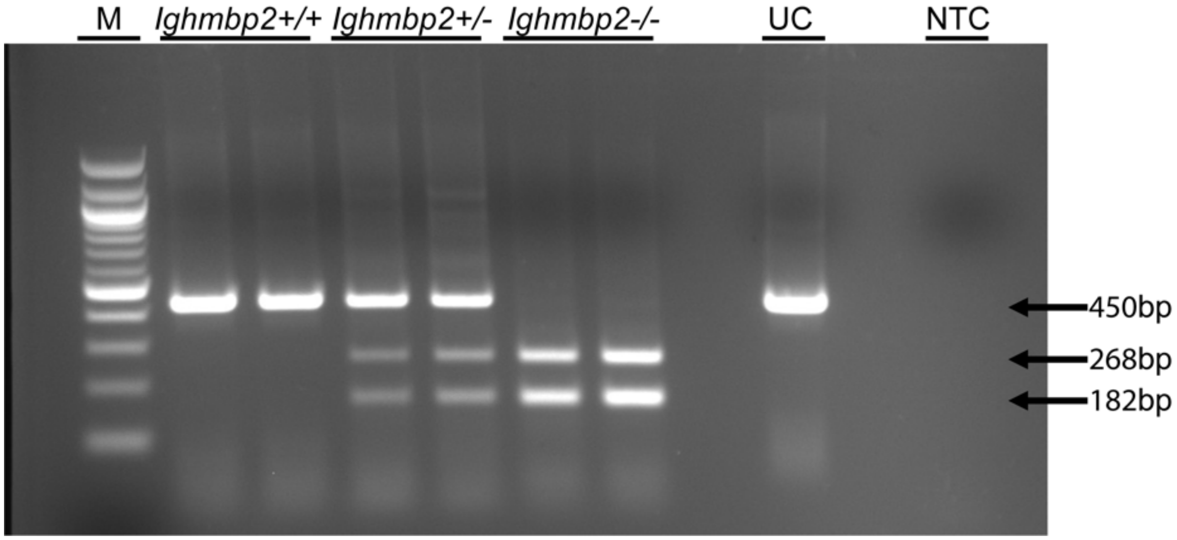

Supplemental Figure 2. Genotyping of wildtype, *Ighmbp2*$^{+/nmd}$ and *Ighmbp2*$^{nmd/nmd}$ mice. The genotype of FVB- *Ighmbp2*$^{+/+}$, *Ighmbp2*$^{+/nmd}$ and *Ighmbp2*$^{nmd/nmd}$ mice was assessed at P1 by means of a tail snip. Genomic DNA was used for PCR and amplicons were digested with *Dde*I and separated on a 2% gel to differentiate wild type from mutant alleles. Controls were undigested sample (UC) and no template control (NTC). M= 100bp DNA marker from New England Biolabs.

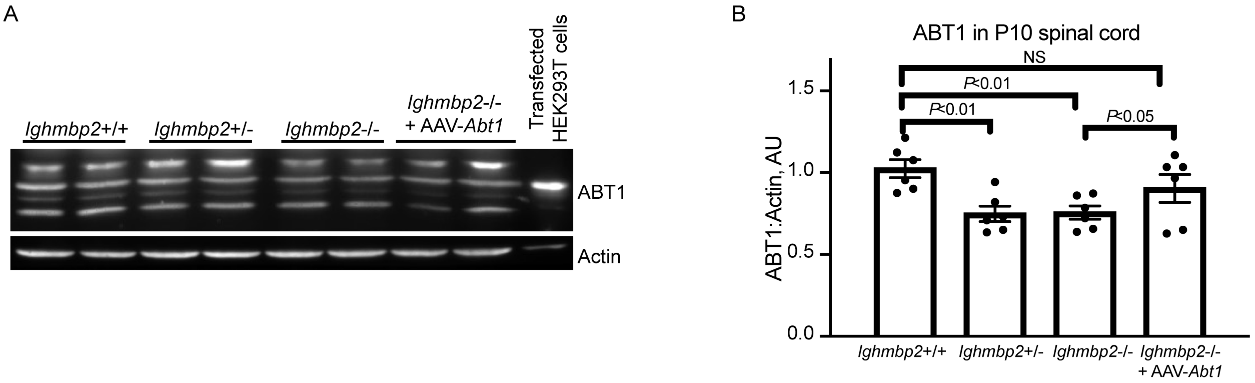

Supplemental Figure 3. ABT1 protein levels are slightly reduced in *Ighmbp2*$^{nmd/nmd}$ mutants but are restored with scAAV9-*Abt1* delivery. P10 spinal cords from (+/+), *Ighmbp2*$^{+/nmd}$, *Ighmbp2*$^{nmd/nmd}$ and *Ighmbp2*$^{nmd/nmd}$ mice injected with scAAV9-*Abt1* (4 X 10^{11} viral genomes) were evaluated. **(A)** Representative western blot. HEK293T cells transfected with scAAV9-*Abt1* served as the ABT1 positive control. Actin served as the loading control. 20μg of protein was added to each lane. **(B)** Quantification of ABT1:Actin based on western blots, *n*=6. ABT1 and IGHMBP2 densitometry data were normalized with actin and analyzed by PROC GLM one-way ANOVA, the error bars represent ± SEM. F-test was used to determine treatment effects and multiple range test for differences between groups. μg=microgram.

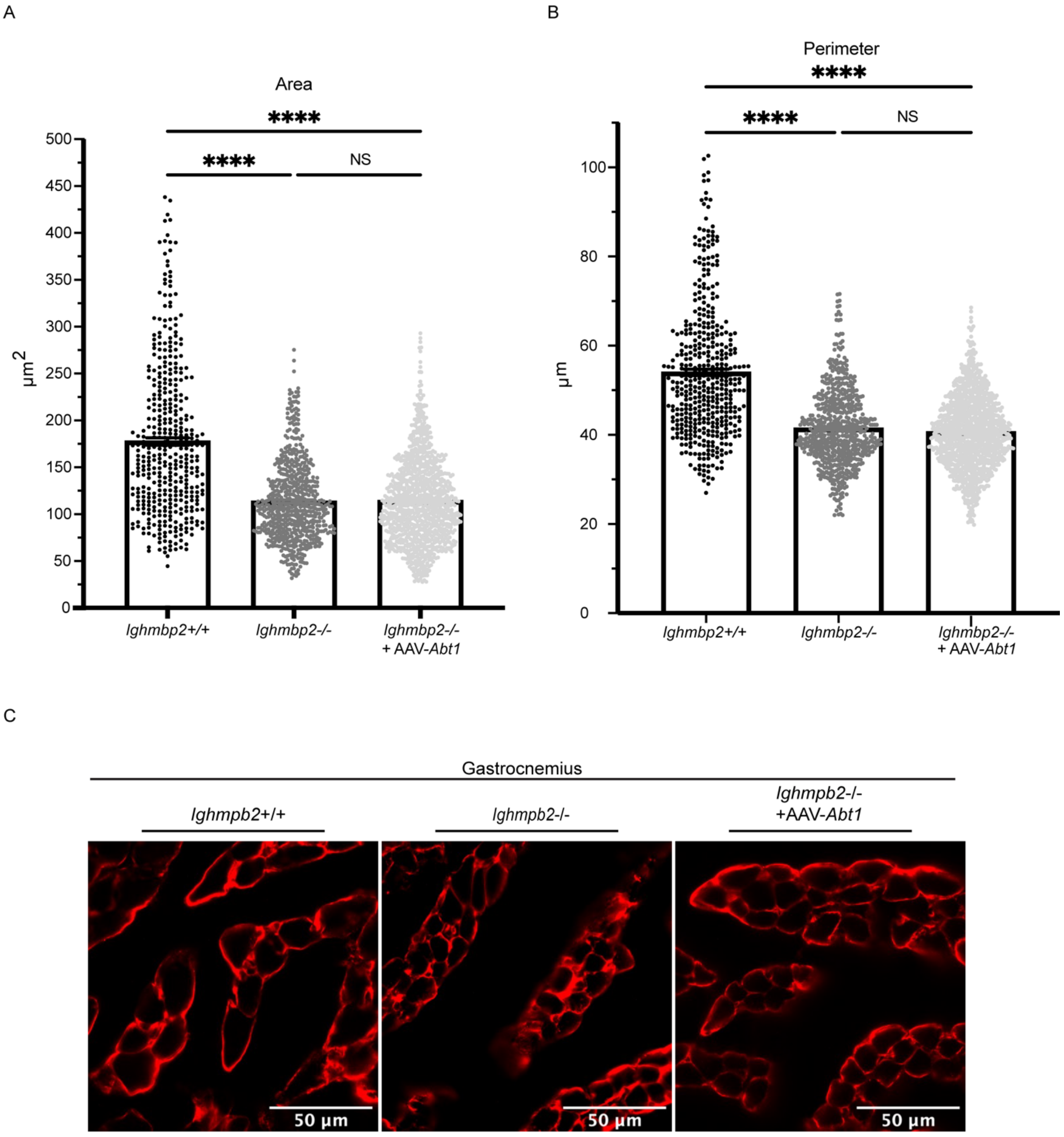
A
Area
****
****
NS
μm2
Ighmbp2+/+
Ighmbp2-/-
Ighmbp2-/-
+ AAV-Abt1
B
Perimeter
****
****
NS
μm
Ighmbp2+/+
Ighmbp2-/-
Ighmbp2-/-
+ AAV-Abt1
C
Gastrocnemius
Ighmpb2+/+
Ighmpb2-/-
Ighmpb2-/-
+AAV-Abt1
50 μm
50 μm
50 μm

Supplemental Figure 4. Muscle fiber area nor perimeter is altered in *Ighmbp2*$^{nmd/nmd}$ mice injected with scAAV9-*Abt1*. (A) Quantification of muscle fiber area of gastrocnemius tissue cross sections. Assessment of P12 wild type (+/+, black), *Ighmbp2*$^{nmd/nmd}$ (dark grey) and *Ighmbp2*$^{nmd/nmd}$ mice injected with scAAV9-*Abt1* (4 X 10^{11} viral genomes) (light grey). **(B)** Quantification of muscle fiber perimeter of gastrocnemius tissue cross sections. Assessment of wild type (+/+, black), *Ighmbp2*$^{nmd/nmd}$ (dark grey) and *Ighmbp2*$^{nmd/nmd}$ mice injected with scAAV9-*Abt1* (4 X 10^{11} viral genomes) (light grey). Statistical significance for A and B was determined by one-way ANOVA with a Tukey's multiple comparison post-*hoc* test, ****=<.0001, data bars expressed as mean ± SEM. *n* > 200 muscle fibers were analyzed and averaged per cohort. Three to four continuous muscle cross sections were obtained per muscle. Muscle fiber area and perimeter measurements were analyzed by manual tracing of myofibers using ImageJ Fiji Software (NIH) in a blinded manner. Statistical analyses were performed with GraphPad Prism software. **(C)** Microscopic images of gastrocnemius tissue sections at P12. Tissue was assessed using anti-laminin antibody. Original magnification 40X.
